# *In Silico* Analysis of Small RNAs Suggest Roles for Novel and Conserved miRNAs in the Formation of Epigenetic Memory in Somatic Embryos of Norway Spruce

**DOI:** 10.3389/fphys.2017.00674

**Published:** 2017-09-08

**Authors:** Igor A. Yakovlev, Carl G. Fossdal

**Affiliations:** Norwegian Institute of Bioeconomy Research Ås, Norway

**Keywords:** conifers, *Picea*, epigenetic memory, epigenetic regulators; miRNAs, somatic embryos

## Abstract

Epigenetic memory in Norway spruce affects the timing of bud burst and bud set, vitally important adaptive traits for this long-lived forest species. Epigenetic memory is established in response to the temperature conditions during embryogenesis. Somatic embryogenesis at different epitype inducing (EpI) temperatures closely mimics the natural processes of epigenetic memory formation in seeds, giving rise to epigenetically different clonal plants in a reproducible and predictable manner, with respect to altered bud phenology. MicroRNAs (miRNAs) and other small non-coding RNAs (sRNAs) play an essential role in the regulation of plant gene expression and may affect this epigenetic mechanism. We used NGS sequencing and computational *in silico* methods to identify and profile conserved and novel miRNAs among small RNAs in embryogenic tissues of Norway spruce at three EpI temperatures (18, 23 and 28°C). We detected three predominant classes of sRNAs related to a length of 24 nt, followed by a 21–22 nt class and a third 31 nt class of sRNAs. More than 2100 different miRNAs within the prevailing length 21–22 nt were identified. Profiling these putative miRNAs allowed identification of 1053 highly expressed miRNAs, including 523 conserved and 530 novels. 654 of these miRNAs were found to be differentially expressed (DEM) depending on EpI temperature. For most DEMs, we defined their putative mRNA targets. The targets represented mostly by transcripts of multiple-repeats proteins, like TIR, NBS-LRR, PPR and TPR repeat, Clathrin/VPS proteins, Myb-like, AP2, etc. Notably, 124 DE miRNAs targeted 203 differentially expressed epigenetic regulators. Developing Norway spruce embryos possess a more complex sRNA structure than that reported for somatic tissues. A variety of the predicted miRNAs showed distinct EpI temperature dependent expression patterns. These putative EpI miRNAs target spruce genes with a wide range of functions, including genes known to be involved in epigenetic regulation, which in turn could provide a feedback process leading to the formation of epigenetic marks. We suggest that TIR, NBS and LRR domain containing proteins could fulfill more general functions for signal transduction from external environmental stimuli and conversion them into molecular response. Fine-tuning of the miRNA production likely participates in both developmental regulation and epigenetic memory formation in Norway spruce.

## Introduction

Adaptation to the changing environments is vitally important for long-lived plant species like forest trees. Epigenetic modifications and specifically epigenetic memory could be important mechanisms for diversifying environmental responses and widening the total plasticity of populations. The epigenetic memory of a plant is defined by the reproducible set of modifications of DNA and chromatin (without alteration of the DNA sequence) induced by external stimuli, which alter gene expression and therefore the properties and behavior of the plant. Memorizing of specific responses, laid down by epigenetic mechanisms, could provide significant strategic benefits to those plants, since the most successful response could be tuned or reenacted in response to a modified environmental condition and this would be retained in future cell lineages, and potentially inherited and altered by selection in future generations (Bräutigam et al., 2013; Baulcombe and Dean, 2014; Iwasaki and Paszkowski, 2014; Kinoshita and Seki, 2014). Much remains to be known about the enigmatic repertoire of epigenetic mechanisms that operate in forest trees but earlier studies firmly confirmed the presence of epitype inducing (EpI) temperature-dependent plant phenotypes (Yakovlev et al., 2012; Liu et al., 2015) and significant transcriptomic changes in such epitypes (Yakovlev et al., 2016).

Both long non-coding RNAs (lncRNAs) and small RNAs (sRNAs) such as short non-coding RNAs are known to be core components of signaling networks involved in epigenetic modification, transcription regulation and participate in transgenerational epigenetic inheritance in plants and animals (Hauser et al., 2011; Heard and Martienssen, 2014). Epigenetic regulation can be mediated through a dynamic interplay between sRNAs, DNA methylation, histone modifications, histone variants, and chromatin architecture, which together modulate transcriptional silencing, activation and the accessibility of DNA in variety of ways (Heo and Sung, 2011; Simon and Meyers, 2011; Lee, 2012; Mirouze, 2012; Bond and Baulcombe, 2014).

MicroRNAs (miRNAs) are specific class of sRNA regulators, with having roles in phenotypic plasticity, plant development and as well as metabolism, all of which act through posttranscriptional regulation of gene expression. miRNAs are widely recognized as having a 20–24 nt length, and characteristically originate from a hairpin-folded single-stranded RNA precursor structure (Meyers et al., 2008). MicroRNA precursors are transcribed from specific miRNA genes (MIR), and are processed predominantly by a multi-functional DICER-LIKE1 (DCL1). The miRNAs in turn negatively regulate gene expression by forming miRNA-inducing silencing complex (miRISC) in association with the ARGONAUTE (AGO) proteins (Bartel, 2004). miRNAs have potential to regulate virtually all cellular mechanisms (Sun, 2012; Wu, 2013), and they do so by restricting translation or cleaving multiple target transcripts. In some instances, they have already been identified as key players in producing rapid adaptation to changing environmental conditions (Sunkar et al., 2012; Ferdous et al., 2015; Nguyen et al., 2015). As miRNAs target transcripts through the imperfect match of sequence composition between miRNA and target, the consequence of gene regulation by miRNAs is that a few miRNAs can specifically change the expression pattern, or fine tune, many specific genes simultaneously. The consequence of gene regulation by miRNAs is similar to that by transcription factors (TFs) (Morris and Mattick, 2014). Moreover, a regulatory cascade essential for appropriate execution of several biological events is triggered through the combinatorial network action of both miRNAs and TFs (Sunkar, 2010; Arora et al., 2013). Besides TFs being among miRNA targets there are known epigenetic regulators (Pikaard and Mittelsten Scheid, 2014), and these in turn, regulate the expression of the individual miRNAs (Gruber and Zavolan, 2013; Song et al., 2015). More specifically, miRNAs are shown to be directly involved in epigenetic regulation and memorizing the responses to different types of stress both in plants and animals (Khraiwesh et al., 2012; Osella et al., 2014; Stief et al., 2014; Hilker et al., 2016). Thus miRNAs have the ability to regulate many target genes, initiate transcriptional and silencing cascades, provide feedback loops, and split transcriptional regulation off into a separate dedicated parallel tracks including those already known to be in the epigenetic regulatory pathway itself.

Conserved and novel miRNAs were identified in angiosperm and gymnosperm species (Montes et al., 2014), including multiple conifers—pines (Lu et al., 2007; Oh et al., 2008; Wan et al., 2012b; Quinn et al., 2014), spruces (Yakovlev et al., 2010; Nystedt et al., 2013), and larches (Zhang et al., 2012, 2013), but their diversity, abundance and regulatory functions are still largely to be studied. Norway spruce is a suitable *Pinaceae* member to conduct experimental studies for epigenetic modification in gymnosperms since a variety of resources exist, including the possibilities for *in vitro* propagation of plant material (Kvaalen and Johnsen, 2008) and draft genome (Nystedt et al., 2013). Furthermore, epigenetic memory in Norway spruce affects vitally important adaptive traits such as the timing of bud burst and bud set, adaptive traits crucial for this species environmental success. Epigenetic alteration of these traits are presumed to be established or modified in response to the EpI temperature conditions prevailing during early seed formation, based phenotypic and molecular responses (Kvaalen and Johnsen, 2008; Johnsen et al., 2009). Moreover, *Picea abies* somatic embryogenesis (SE) is an ideal experimental system for studying this phenomenon since these responses are recapitulated through *in vitro* SE. SE at different temperatures closely mimic the processes of epigenetic memory formation, naturally occurring in zygotic seeds, and give rise to epigenetically different plants (epitypes), which have a clonal character, with a reproducible and predictable temperature-dependent altered bud phenology.

Some progress has already been achieved toward understanding molecular mechanisms underlying the epigenetic memory formation. A significant number of epigenetic regulators, including sRNA biogenesis pathways, are differentially expressed at different EpI conditions, supporting that methylation of DNA, histones modifications and sRNAs are pivotal for the establishment of the epigenetic memory (Yakovlev et al., 2014, 2016). We found several miRNAs differentially expressed in buds of different epitypes and suggesting their involvement in the epigenetic memory (Yakovlev et al., 2010), and this stimulated the need for a much deeper study of the various miRNA during SE in spruce, the life stage at which the epigenetic memory is laid down. The main goal of the current study was to further characterize and identify the extensive miRNA fraction in the small non-coding part of Norway spruce transcriptome. We aimed to identify the key miRNAs regulating differentially expressed genes (DEGs) and those especially related to epigenetic regulators that could potentially establish the epigenetic memory process during embryogenesis, by examining their expression profiles during SE at contrasting EpI temperature conditions. To our knowledge, this is also the first genome-wide *in silico* characterization of miRNAs and study of their transcript profiles during SE in spruce. We show an extensive number of miRNAs that can target epigenetic regulators including those modifying DNA and histone methylation, and sRNA pathways genes, supporting the notion that these predicted miRNAs and their target genes could be among central players in epigenetic memory formation.

## Methods

### Plant material and RNA extraction

Somatic embryos, and their induction and growth, used in this analysis are already previously described (Kvaalen and Johnsen, 2008; Yakovlev et al., 2014, 2016). Embryogenic samples were those obtained from two seeds (genotypes) originating from a controlled cross of a defined female (♀#2650) and male (♂#2707) of Norway spruce parents, with those crosses being performed either in outdoor conditions; a cold originated genotype, denoted as A2C, or in greenhouse conditions as a warm originated genotype, denoted as B10W. With the current analysis, nine samples were collected from each of the two different genotypes, representing three maturation stages and three different temperatures in which the epitypes form, providing 18 samples in total. Twenty to Thirty milligram of embryo containing callus or 2–5 embryos were collected per sample, combined and immediately snap-frozen and stored in liquid nitrogen until RNA extraction. Embryo tissues were ground in 1.5 ml Eppendorf tubes by pestle and the total RNA extracted using an Epicentre MasterPure™ Plant RNA Purification Kit (Epicentre, Madison, WI, USA, #MCR85102) according to the manufacture's instruction. Total RNA preparations were stored at −80°C and the integrity and quantity of total RNA was assessed by Agilent 2100 Bioanalyzer with RNA 6000 Nano Kit and also the Small RNA kit (Agilent, Santa Clara, CA, USA #5067-1511 and #5067-1548 respectively).

### Small RNA library construction, sequencing and bioinformatics

The 18 small RNA libraries were each constructed from 1 μg of total RNA, using the Ion Total RNA-Seq Kit v2 for Small RNA Libraries (#4476289), with the enrichment steps as outlined in the Ion RNA-Seq Library Preparation guide (#4476286 revision E). Quality and quantity of amplified libraries were analyzed with the Agilent Technologies 2100 Bioanalyzer with Agilent High Sensitivity DNA Kit (Agilent, Santa Clara, CA, USA, #5067-4626). Template-positive Ion Sphere™ Particles (ISPs) containing clonally amplified DNA were prepared with the Ion OneTouch™ 2 Instrument using the Ion PGM Template OT2 200 Kit (#4480974) according manufacturer instructions. Sequencing was performed using Ion Personal Genome Machine® (PGM™) Sequencer (Thermo Fisher Scientific Inc.) and each library was sequenced individually on 316v2 chips using the Ion PGM™ sequencing 200 Kit v2 (#4482006). Data was initially processed on Ion Torrent Server using Torrent Suite software (v.4.2) and fastq formatted files were analyzed using CLC Genomics Workbench software (V 8.+) (QIAGEN, Aarhus A/S, Denmark).

### *In Silico* identification of conserved and novel mirnas and miRNA genes in spruce

All the single-read and sRNA sequences beyond 19–29 bp from the 18 libraries were filtered out to remove rare and too short or too long reads. To search for conserved miRNAs, the filtered sRNA sequences were compared to known miRNAs in the miRBase v21.0 (Kozomara and Griffiths-Jones, 2011) restricted to all tree species in addition to miRNAs from the genomes of *Arabidopsis, Vitis*, and *Physcomitrella* allowing up to 2 nucleotides mismatch using the sRNA module of CLC genomics workbench software (v.8). To identify miRNA gene loci and novel miRNAs, we mapped all the filtered sRNA reads to the gene models encompassing high-, medium- and low-confidence as defined in the *Picea abies* genome v1 (http://congenie.org/) (Nystedt et al., 2013). A gene model was considered a putative miRNA gene loci when at least 100 reads of distinct sRNA tags mapped to the loci or gene model with a minimum of 0–2 mismatches. Gene sequences having 80–300 nt flanking the candidate miRNA sequence were manually scrutinized based on the criteria for miRNA definition described by Meyers et al. (2008). In addition, secondary structures of putative miRNA genes were predicted using different folding algorithms by the CLC genomics workbench software. When the stem-loop hairpin for the putative MIR was confirmed, then the existence of putative novel miRNAs was estimated. sRNA(s) with higher frequency was/were considered as guide miRNAs, sRNA(s) on the opposite strand of the loop was/were considered as star-miRNAs (^*^miRNA). We allowed shifting of star sequences relative to miRNA guide sequences for 1–6 bp.

During analysis, we established that some miRNA candidates (guide and star sequences) were determined to have considerably long hairpin structures, so we extended our search for gene models within the fragments for up to 1 kb, using the same procedure for miRNA detection as those used in the shorter fragments.

### Expression analysis of predicted miRNAs and *in Silico* identification of their targets

Expression analysis was performed using RNA-Seq tool of the CLC Genomic Workbench v8 with defined lists of miRNAs for annotation of the sRNA reads. Prediction of miRNA targets was carried out by searching for complementary regions between the identified miRNAs in this study and by using all the *Picea abies* gene models v1 as the transcript sequence input using online web server psRobot—Plant Small RNA Analysis Toolbox (Wu et al., 2012), and the psRNATarget—Plant Small RNA Target Analysis Server (Dai and Zhao, 2011).

To substantiate putative targets and to refine potential miRNA-mRNA target pairs, we additionally analyzed correlations between transcript amounts of miRNA and their defined targets at three different EpI temperatures. mRNA transcript amounts were taken based on our previous study (Yakovlev et al., 2016).

### qRT-PCR for miRNAs

To validate sequencing data we quantified transcript levels for 10 selected conserved and novel miRNAs with quantitative real-time RT-PCR. For analysis we used the same small RNA extracts which were used for sequencing. cDNAs were synthesized from 600 ng of the small RNA extracts with the Mir-X™ miRNA First-Strand Synthesis kit (Clontech, #638315) following manufacturer recommendations. Real-time RT-PCR amplification was performed using Mir-X™ miRNA qRT-PCR SYBR® Kit (Clontech, #638314) in a 25 μl reaction volume, using 2 μl of a diluted cDNA solution described above as template and 200 nM of each primer. qPCRs were performed on a ViiA™ 7 Real-Time PCR System (Applied Biosystems, USA) following the manufacturer's instructions. After PCR, dissociation curves were carried out to verify the specificity of the amplification. There were three biological replicates for each sample. All expression levels were normalized to geometric mean of three selected ribosomal and transfer RNA genes (Pa4.5S, Pa5S and PatRNA-R), showing most stable expression profiles as describe previously (Yakovlev et al., 2010). Forward primers were designed based on mature miRNA sequence. If Tm of mature miRNA was less than 60°C, it had been adjusted by adding G's to the 5′ end of the miRNA sequence. The list of studied miRNAs and their primer sequences are shown in Table S10. The 3′ primer for qPCR was the mRQ 3′ Primer supplied with the kit.

Data acquisition and analysis were done using ViiA™ 7-system SDS software for absolute quantification and MS Excel software.

### Data submission

Unique transcripts from 18 libraries sequenced using Ion Torrent PGM™ Sequencer were deposited to the SRA (Short Read Archive, NCBI) and got the following accession: submission ID SUB1781210; BioProject ID PRJNA339513 and accession IDs: SAMN05592191–SAMN05592208.

## Results

### Small RNA library sequencing

**Figure 1:**
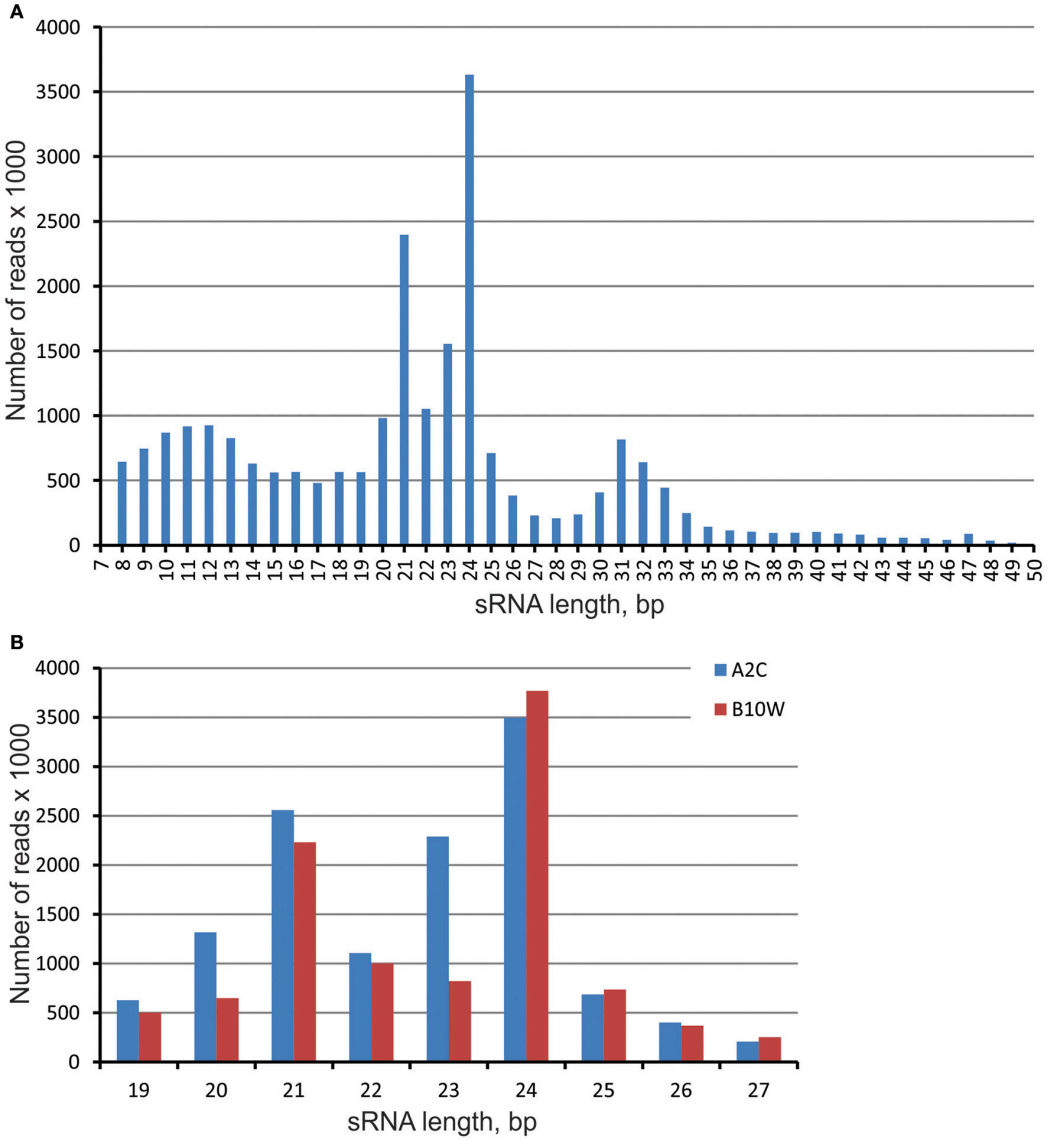
Length distribution of sRNA in libraries prepared from the embryos of two genotypes of the full-sib family of Norway spruce from cross ♀#2650 × ♂#2707 produced in outdoor conditions—A2C and produced in greenhouse conditions—B10W. **(A)** averaged sRNA length distribution in all libraries; **(B)** sRNA length distribution in miRNA length range—19–27 bp in two genotypes.

In total, we sequenced 18 small RNA (sRNA) libraries, representing three stages of *in vitro* spruce embryo development and three different EpI temperature treatments. This produced nearly 50 million reads in the length range from 7 to 50 nt (Table S1). Three clear read length peaks were found in the embryonic sRNA pool after the trimming—and these corresponded with the lengths of 24–23, 21–20, and 31–32 nt (Figure 1A). The 31–32 nt group of sRNAs consist of ~14,5 thousands sRNAs among more than 3 million reads. To reduce complexity and focus attention on the canonical miRNA population, we filtered all reads to 19–27 nt and removed all single reads to avoid sequencing and stochastic errors. In total, over 13 million reads were retained for further analysis with two clear peak classes—prevailing with length of 24–23 nt and then of 21–20 nt, in both genotypes A2K and B10W (Figure 1B).

### *In Silico* identification of conserved and novel miRNAs in spruce

Using CLC Genomics Workbench a sRNA analysis was conducted. Search and annotation of conserved miRNAs was based on the miRBase v21 database using a criteria allowing up to two mismatches. A total of 636 conserved miRNAs were defined and these belonged to 51 miRNA families. These could originate from 99 defined precursors. Not all precursors for the conserved miRNA members were found, but at least one precursor was identified for the majority of miRNA families and we considered that sufficiently supported the internal origin of the defined class of conserved miRNAs (Table S2).

Additionally we defined 1316 novel miRNAs that had no homology to miRBase v21 annotations. They could belong to 630 families and could originate from 740 predicted precursors (Table S3).

**Figure 2:**
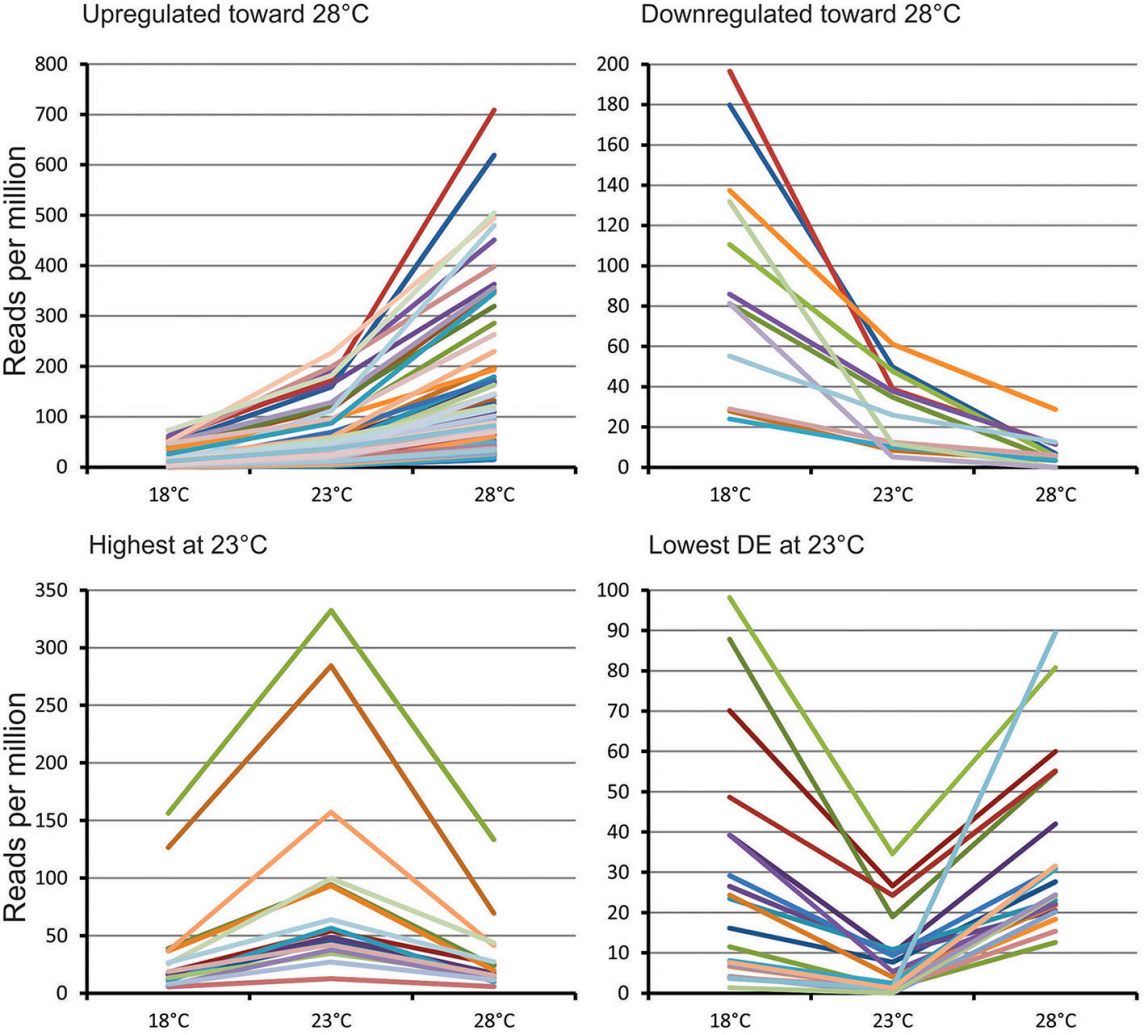
Main expression profiles of predicted differentially expressed miRNAs in Norway spruce embryos cultivating at three different EpI temperatures.

The majority of identified miRNAs in spruce embryos were 21 nt (41%) and 22 nt (34%) in length, all other length classes count less than 10% (Figure 2) (Figure S1). More often the miRNAs at the initial positions contain uridine (U–37%) and adenine (A–25%) and less C and G (16 and 22%% correspondingly).

For nearly all conserved miRNA families we found large numbers of miRNA members (isomiRs). In average, there were 15 isomiRs per family, but varied from 1 to 102. The largest quantity of isomiRs was found for the highly conserved family miRNA166, with 102 miRNAs. More than 40 miRNAs were found across the identified miRNA families—of miR156, miR159, miR946, miR950, miR951, miR1311 and miR3701. Eleven families had more than 10 but less than 40 member miRNAs and these included miR319, miR390, miR396, miR397, miR482, miR947, miR1312, miR1316, miR3705, miR3710, and miR3712. The remaining 39 families had less than 10 isomiRs (Table 1).

**Table 1:** Family description, targets and putative functions of predicted conserved miRNAs families defined in within the sRNAs of Norway spruce embryos in comparison with previously published data.

	**Conserved miRNA family**	**Number of miRNAs (isoforms)**	Number of highly expressed miRNAs^*^	**Number of putative target gene models**	**Targets of miRNAs defined in our study**	**Described and putative targets of miRNAs**	**Putative functions**	**References**
1	Pab-miR1311	41	29	6	DnaJ domain proteins, NBS-LRRs	unknown	Environmental signal transduction and response mechanisms; gymnosperm-specific	Wan et al., 2012b
2	Pab-miR1312	19	7	12	TIR domain; NBS-LRRs; AAA proteins	Argonaute/ Zwille-like family; Flagellin-sensing 2-like family	Involved in miRNA biogenesis pathway regulation, mediates the innate response to bacterial pathogens	Wan et al., 2012b
3	Pab-miR1313	5	2	9	Protein tyrosine kinase with LRR domains	unknown	Cellular signaling pathways, gymnosperm-specific	Wan et al., 2012b
4	Pab-miR1314	9	4	11	Unknown; RNA polymerase; transcription factor ICE1-like	Brassinosteroid Insensitive 1-LRR receptor Serine/Threonine kinase	extracellular stress signal transduction, mediate the response to brassinosteroid (BR) hormones	Wan et al., 2012b
5	Pab-miR1315	4	4	39	DRF autoregulatory domain; LRR domains	unknown	Part of signaling mechanisms, upstream GTPase signals to regulate cellular processes - cytokinesis, cell polarity, and organelle motility; gymnosperm-specific	Wan et al., 2012b
6	Pab-miR1316	10	6	26	Ribosomal protein S7p/S5e; Region in Clathrin and VPS; Lipase (class 3)	unknown	vesicular sorting and trafficking pathways and essential for body plan development, defense and response to the environment; gymnosperm-specific	Wan et al., 2012b
7	Pab-miR156	62	44	56	SBP domain; Glycosyl hydrolases family 16	Squamosa promoter-binding protein (SBP)-like family	Tissues development and maturation	Huang et al., 2013
8	Pab-miR159	52	23	9	MYB family; Plant transcription factor NOZZLE; bZIP transcription factor	MYB family	Floral initiation and anther development; seed germination, GA and ABA signaling pathways	Reyes and Chua, 2007; Zhang et al., 2010
9	Pab-miR160	5	3	No DEGs	–	Auxin response factors - ARF	Modulates expression of auxin-response genes during tissues development, connected with miR167	Liu et al., 2007
10	Pab-miR162	7	5	No DEGs	–	DCL1-like	DCL1 miRNA biogenesis transcriptional feedback loop	Zhang et al., 2012
11	Pab-miR164	1	–	No DEGs	–	NAC domain containing family	polar auxin transport (PAT) and transduces auxin signals to promote root development	Lu et al., 2015
12	Pab-miR165	2	1	21	Homeobox; bZIP transcription factor; Exostosin; Protein kinase	Class III HD-ZIP TFs	polar auxin transport and maintenance of shoot apical meristem and vascular patterning	Huang et al., 2013; Barik et al., 2014
13	Pab-miR166	102	74					
14	Pab-miR167	9	6	2	Auxin response factors - ARF	Auxin response factors - ARF	control of stress/ temperature and auxin responsive signaling during development, phenotypic plasticity, somatic embryogenesis	Wu et al., 2006; Burklew et al., 2014
15	Pab-miR168	3	2	No DEGs	–	Argonaute family (AGO1 YA) family	AGO1-mediated post-transcriptional silencing, virus resistance	Jagtap and Shivaprasad, 2014
16	Pab-miR169	9	3	1	CCAAT-binding transcription factor	CCAAT-binding transcription factor (CBF-B/NF-YA) family	Abiotic stress response, ABA signaling pathways	Zhang et al., 2010
17	Pab-miR171	8	4	1	GRAS family transcription factor	SU(VAR)3-9 homolog8; scarecrow-like (SCL) and GRAS transcription factors	Response to exogenous auxin and very early cellular differentiation and development	Zhang et al., 2010; Wan et al., 2012b
18	Pab-miR172	3	3	4	AP2 domain; unknown	apetala2 - AP2	Seed development and maturation, response to cold	Zhang et al., 2010
19	Pab-miR2118	1	-	No DEGs	–	Anion exchanger family - AE1	Disease resistance	Wan et al., 2012b
20	Pab-miR2916	6	2	No DEGs	–	unknown	–	Huang et al., 2013
21	Pab-miR319	25	15	No DEGs	–	Myb and TCP family transcription factors	versatile functions in multiple aspects of plant growth and development and abiotic stress response	Reichel and Millar, 2015
22	Pab-miR3630	2	-	No DEGs	–	LRR receptor-like family kinase; auxin-responsive gh3 family	multiple functions in plant growth and development	Pantaleo et al., 2010; Boke et al., 2015
23	Pab-miR3693	3	1	No DEGs	–	NBS-LRR family	Extracellular signal transduction, disease resistance	Yakovlev et al., 2010
24	Pab-miR3694	4	2	No DEGs	–	Unknown	–	Yakovlev et al., 2010
25	Pab-miR3695	4	1	No DEGs	–	Unknown	–	Yakovlev et al., 2010
26	Pab-miR3696	5	4	No DEGs	–	Unknown	–	Yakovlev et al., 2010
27	Pab-miR3697	6	5	19	TIR domain; NBS-LRR proteins; BRCA2 repeat	NBS-LRR family	Extracellular signal transduction, disease resistance	Fossdal et al., 2012
28	Pab-miR3699	4	1	1	Unknown proteins	Unknown	–	Yakovlev et al., 2010
29	Pab-miR3700	5	4	1	Unknown proteins	Unknown	–	Yakovlev et al., 2010
30	Pab-miR3701	50	26	27	NBS-LRR proteins; Cellulose synthase	Unknown	–	Yakovlev et al., 2010
31	Pab-miR3702	4	3	No DEGs	–	SPT4	involved in RNA polymerase V -mediated transcriptional gene silencing	Yakovlev et al., 2010; Köllen et al., 2015
32	Pab-miR3703	8	7	2	MutS domain V mismatch repair	Unknown	gymnosperm-specific	Yakovlev et al., 2010; Köllen et al., 2015
33	Pab-miR3705	10	5	1	Pyridine nucleotide-disulphide oxidoreductase	NBS-LRR family	Extracellular signal transduction, disease resistance	Fossdal et al., 2012
34	Pab-miR3706	4	3	3	Protein tyrosine kinase; Leucine Rich Repeat; ABC1 family	Unknown	Stress signaling; gymnosperm-specific	Yakovlev et al., 2010
35	Pab-miR3707	6	6	14	TIR domain; NBS-LRR proteins	Unknown	Stress signaling; gymnosperm-specific	Yakovlev et al., 2010
36	Pab-miR3708	5	5	No DEGs	–	Unknown	–	Yakovlev et al., 2010
37	Pab-miR3709	9	4	5	NBS-LRR proteins	Unknown	Stress signaling; gymnosperm-specific	Yakovlev et al., 2010
38	Pab-miR3710	21	17	256	NBS-LRR proteins; DRF autoregulatory domain; IPP transferase; Rdx family	Unknown	Stress signaling; gymnosperm-specific	Yakovlev et al., 2010
39	Pab-miR3711	3	2		–	Unknown	–	Yakovlev et al., 2010
40	Pab-miR3712	13	8	9	TIR domain; NBS-LRR family; AAA domain	Unknown	–	Yakovlev et al., 2010
41	Pab-miR390	10	5	No DEGs	–	TAS3 transcripts	Ta-siRNA biogenesis, developmental timing and patterning	Yoshikawa, 2013
42	Pab-miR394	4	3	4	hAT family dimerisation domain; LRR domains	LCR (Leaf Curling Responsive-ness) F-box family	leaf morphology, regulation of the cell cycle, response to abiotic stresses	Knauer et al., 2013
43	Pab-miR396	35	14	12	F-box family; UDP-glucosyl transferases;	growth-regulating factors (GRFs)	cell proliferation	Rodriguez et al., 2010; Knauer et al., 2013
44	Pab-miR397	11	2	21	Multicopper oxidases	ATP sulfurylase (ATPS)	involved in oxidative stress response, photosynthesis, and cellular respiration, mediating copper homeostasis	Burklew et al., 2014
45	Pab-miR398	1	1	No DEGs	–	copper/zinc superoxide dismutase (SOD)		
46	Pab-miR399	1	–	No DEGs	–	phosphate/E2 ubiquitin-conjugating family (PE2U)	maintaining phosphate homeostasis, temperature dependent regulation of flowering	Kim et al., 2011; Burklew et al., 2014
47	Pab-miR408	3	2	1	unknown	Laccase 3; thiamin pyrophosphokinase1	mediating copper homeostasis, abiotic stress responses, alkaloids biosynthesis	Boke et al., 2015; Ma et al., 2015
48	Pab-miR4414	1	1	No DEGs	–	Tetratricopeptide repeat (TPR)-like superfamily	Protein interactions during transcription and protein transportation	Wang et al., 2011; Liu et al., 2014
49	Pab-miR482	39	26	54	NBS-LRR family; Myb-like DNA-binding domain	NBS-LRR disease resistance family; Histone deacetylase	Signal transduction, resistance to disease or other stresses	Shivaprasad et al., 2012
50	Pab-miR535	7	5	4	ACT domain with acetolactate synthase	Squamosa promoter-binding family –like – SPL2; DNA/RNA helicase family	low temperature-responsive miRNA, regulation of timing of transition from vegetative to reproductive phase	An et al., 2011
51	Pab-miR6478	4	2	No DEGs	–	Protein of unknown function (DUF3537)	Unknown	Liu et al., 2014
52	Pab-miR828	2	-	No DEGs	–	TAS4 transcript; MYB-like and WER TFs	Ta-siRNA biogenesis, regulation of transcription, stress response	Guan et al., 2014
53	Pab-miR946	46	31	27	NBS-LRR family; RING-type zinc-finger; HEAT repeats	Unknown	involved in extracellular signal transduction, protein bindings and intracellular transport	Wan et al., 2012b
54	Pab-miR947	10	5	3	Unknown	Unknown	–	Wan et al., 2012b
55	Pab-miR948	3	3	4	Protein tyrosine kinase	Unknown	modulates enzymatic activity and creates binding sites for the recruitment of downstream signaling proteins	Wan et al., 2012b
56	Pab-miR949	7	5	6	Unknown	Unknown	–	Wan et al., 2012b
57	Pab-miR950	77	51	196	TIR domain; NBS-LRR family; AP2 domain; 50S ribosome-binding GTPase	Cytoplasmic ribosomal family S13-like; NBS-LRR family	Extracellular signal transduction, disease resistance	Wan et al., 2012b
58	Pab-miR951	42	25	34	TIR domain; NBS-LRR family; Cytochrome P450 family	NBS-LRR family	Extracellular signal transduction, disease resistance	Fossdal et al., 2012
		851	522	901				

* *With more than 10 reads*.

### Quantification of transcripts and identification of differentially expressed miRNA profiles

For expression analysis, we used all miRNAs with average read counts greater or equal to 10 in at least one of the sequenced libraries. From the 2267 miRNAs we defined in the transcriptome of Norway spruce embryos, 1115 miRNAs were further used for differential expression analysis.

Differentially expressed miRNAs (DEMs) were identified through pair-wise comparison of libraries by setting the threshold |log_2_ RPKM ratio| to more than 1 and *p*-value < 0.05. Temperature responsive miRNAs displaying more than two fold difference between EpI temperatures were considered as differentially expressed and these were further examined. In total, we detected 676 DEMs while the remaining 439 miRNAs, did not show any differences in transcript numbers at different EpI temperatures. Most of the defined miRNAs were present in all treatments. Among the DEMs, only one miRNA—Pab-miRn931 was expressed solely at 28°C and 15 miRNAs were expressed at two temperatures and were absent at third particular temperature.

Based on their transcript profiles at the three different EpI temperatures, 654 DEMs could be grouped into 12 clusters. Main transcription profiles shown on Figure 1 and detailed description of clusters presented on Table S4. The first two clusters included 159 miRNAs significantly upregulated at 28°C and decreasing in abundance with decreasing the temperature. The most abundant here were conserved miRNAs from miR156, miR159, miR166, miR167, miR396, miR1311, miR3701, and miR951, as well as 26 novel miRNAs. Two other clusters (5–6) contained 50 miRNAs significantly upregulated at 18°C and decreasing in abundance with increasing temperature. Most of miRNAs in this cluster were novel, and only conserved miRNAs from miR950 and miR482 families were identified based on sequence similarities. Two clusters (10–11) encompass 22 miRNAs found most abundant at 23°C (Figure 1). Two conserved miRNAs from miR319 and miR3701 families were found here, yet all the other discovered miRNAs were found to be novel. Other clusters consisted of various other miRNAs having similar transcript profiles, the largest of which were clusters 3 and 4. Cluster 3 encompassed 101 miRNAs that were highly expressed at 28°C, yet were of equally low expression at 18° and 23°C, while cluster 4 had an opposing profile, and this encompassed 151 miRNAs showing equal high expression at 28° and 23°C, while showing low expression at 18°C. Both clusters include different miRNAs from the families of miR156, miR159, miR166, miR167, miR396, miR946, miR1311, miR1312, miR3701 miR951 as well as many novel miRNAs (Table S4).

Additionally, we specifically analyzed the changes in sequence and abundance of DEMs from conserved families at different EpI temperatures. Within each family, we found wide range of modifications, including nucleotide substitutions, 5′ and 3′ uridylation and adenylation, trimming and tailing. In addition, we found quite variable transcription patterns for different family members, sometimes opposite. However, we did not find any EpI temperature specific isomiRs presented only at one specific culturing temperature and did not find any clear influence of EpI temperature on modification type. Some examples of miRNA diversity within families, their expression patterns and their corresponding stem-loop RNA secondary structure of hairpin-forming precursors presented at Figure S2.

### *In Silico* prediction of targets of conserved and novel norway spruce miRNAs

For the assignment of functional roles to the whole set of defined miRNAs, the target gene transcripts were predicted by the online web server psRobot—Plant Small RNA Analysis Toolbox (Wu et al., 2012) and the psRNATarget—Plant Small RNA Target Analysis Server (Dai and Zhao, 2011). In the first instance, we searched for the respective target genes for all miRNAs as defined in Norway spruce v1 coding sequences (Nystedt et al., 2013) irrespective of their transcript profiles. This resulted in 2050 miRNAs being identified as the cognate miRNAs to 6058 annotated gene models from around 1414 gene families with diverse biological functions and 4701 gene models without matches to the database. The largest number of gene models, which could be regulated by miRNAs, were in gene families containing following Pfam domains: Leucine Rich Repeat (LRR) protein genes, protein kinase domain, pentatricopeptide (PPR) repeat, NB-ARC (nucleotide-binding adaptor R-gene shared) domain, ATPase family associated with various cellular activities (AAA), Toll-Interleukin receptor (TIR) domain, Clathrin heavy chains/VPS (vacuolar protein sorting-associated), tetratricopeptide (TPR), Myb-like DNA-binding domain, mTERF (Mitochondrial transcription termination factor), Multicopper oxidase, AP2 domain, Cytochrome P450, F-box domain and many others (Table S5). TIR and NBS-LRR comprise one of the largest groups of genes in spruce. We found more than 1900 gene models containing different LRR domains and more than 740 gene models containing NB-ARC domain and close to 370 models containing TIR domain. In total, 2594 for the TIR or NB-ARC LRR gene models were found.

### Differential expression of miRNAs and their predicted targets

Afterwards we selected putative targets among DEGs for the 522 conserved and 593 novel DEMs under the inductive conditions required of epitype differentiation. We additionally analyzed correlations between miRNA and their target transcription patterns at different EpI temperatures to refine potential cognate miRNA-mRNA target pairs. All target pairs with correlation below −0.6 were considered as prospective miRNA regulated gene models, especially as correlations between target transcript and miRNAs at all temperatures could help build a robust definition functional pairs, providing further insight into temperature-dependent processes leading to the formation of epigenetic memory in developing embryos (Tables S6–S8).

In total, we defined 1921 miRNA—mRNAs (DEM–DEG) pairs, consist of 470 miRNAs and 1139 target genes, incl. 930 annotated gene models from around 212 gene families with diverse biological functions and 209 gene models without match at the NCBI databases. Similar to the whole set of miRNAs, the largest number of DEM/DEG pairs were found in gene families coding for tandem repeat domain (TRD) containing proteins. Among the gene families are the 166 LRR gene models, which could be targeted by 278 miRNAs; 90 NB-ARC—by 169 miRNAs, and 52 TIRs—by 138 miRNAs. TIR, NBS-LRR proteins could be targeted by both conserved (miR482, miR946, miR950, miR1311-1316, miR3710, etc.), and novel miRNAs, like Pab-miRnY45_str, Pab-miRn00543, Pab-miRn00468, Pab-miRn00930, Pab-miRn00202_3p, Pab-miRn00386, Pab-miRn00492, Pab-miRn00930, Pab-miRn01804_5p, Pab-miRnB5, etc. Clathrin and vacuolar protein sorting (VPS) domain proteins often contain PPR and TPR repeat domains, and these could be the targets for regulation by miRNAs based on duplex sequence similarity. 30 Clathrin/VPS and 128 PPR/TPR gene models could be targeted by 202 miRNAs, including Pab-miRn00676, which could regulate the translation of 43 genes (Table 2; Table S6).

**Table 2:** Enrichment of Pfam domains based on the preliminary functional characterization of most abundant differentially expressed gene families, which could be regulated by highly differentially expressed miRNAs.

**Pfam ID**	**Pfam Description**	**Number of targeted genes models**	**Number of miRNAs**
PF00560	Leucine Rich Repeat (LRR)	163	274
PF00069	Protein kinase domain	97	139
PF00931	NB-ARC (nucleotide-binding adaptor R-gene shared) domain	90	169
PF01535	Pentatricopeptide (PPR) repeat	84	105
PF01582	Toll-Interleukin receptor (TIR) domain	52	138
PF00004	ATPase family associated with various cellular activities (AAA)	52	85
PF00515	Tetratricopeptide (TPR) repeat	44	57
PF00637	Clathrin heavy chain/VPS (vacuolar protein sorting-associated)	30	40
PF00394	Multicopper oxidase	15	44
PF02536	mTERF (Mitochondrial transcription termination factor)	11	15
PF00646	F-box domain	11	11
PF00249	Myb-like DNA-binding domain	10	12
PF00847	AP2 domain	8	8
PF01397	Terpene synthase, N-terminal domain	8	8
PF00418	Microtubule-associated protein (MAP) Tau, tubulin-binding repeat	5	7
PF06345	DRF (Diaphanous-related formins) autoregulatory domain	4	11
PF00566	Rab-GTPase-TBC domain	4	5
PF01715	tRNA Delta(2)- isopentenylpyrophosphate transferase (IPP transferase)	3	15
PF00046	Homeobox domain	3	11
PF00201	UDP-glucoronosyl and UDP-glucosyl transferase	3	5
PF08263	Leucine rich repeat N-terminal domain	3	4
PF11721	Di-glucose binding within endoplasmic reticulum	3	4
PF04937	Protein of unknown function (DUF 659)	3	3
PF08744	Plant transcription factor NOZZLE	3	3
PF03110	SBP (SQUAMOSA promoter binding protein-like) domain	2	40
PF00106	short chain dehydrogenase	2	7
PF00514	Armadillo/beta-catenin-like repeat	2	5
PF00227	Proteasome subunit	2	4
PF01764	Lipase (class 3)	2	4
-	Pfam domains families with less than 3 genes, total	211	
-	Not annotated gene models	209	
	Total number of target gene models	1,139	

DEMs were also showed to target several transcription factors such as Squamosa promoter-binding (SPB) protein (target of miR156), plant transcription factor NOZZLE-like (miR159), Myb-like (miR159, miR482), Homeobox domain bZIP transcription factor (miR166), HD-ZIP protein (miR165/miR166), CCAAT-binding transcription factor (miR169) and AP2-like transcription factors (miR172, miR950), involved in developmental timing and transition from juvenility. Besides transcription factors, other targets included F-box protein (miR396), laccase (miR397), plant U-box (PUB) proteins (miR946).

We found that 317 DEGs could be regulated by several miRNAs and 290 DEMs could regulate more than one gene (Figures S3.1–20). For example, six DEMs- Pab-miR950.59, Pab-miR950.67, Pab-miR950.68, Pab-miR950.69, Pab-miR950.70 and Pab-miR950.72 from the miR950 family could target the same gene model MA_10433003g0010, coding for multi-domain TIR-NBS P-loop containing Nucleoside Triphosphate Hydrolase domain and involved in signal transduction and response to different external stimuli (Figure S3.1). Pab-miRn0282.1, Pab-miRn0054.4_5p, Pab-miR950.59, and Pab-miR950.72 could target the same gene model MA_55143g0010 at four different target sites (Figure S3.2). MA_55143g0010 is coding for multi-domain TIR-NBS-LRR protein, containing Helix-hairpin-helix motif and probably in addition to signal sensing, monitoring and transduction functions could directly bind to DNA and could fulfill “reader” functions.

Similarly, the novel Pab-miRn0539_5p was found to potentially regulate 12 different gene transcripts of NBS-LRR and ATPases; while conserved Pab-miR1315.1 could regulate 9 genes, mostly LRRs and cytoskeleton remodeling proteins participating in regulation of cellular processes such as cytokinesis, cell polarity, and organelle motility. In most cases, such redundant targeting occurs in gene families of repeat proteins, such as multiple multi-domain TIR-NBS-LRR proteins, proteins kinases, HEAT-repeat proteins and tetra- (TPR) or pentatricopeptide (PPR) repeat proteins (Figures S3.4,5).

### *In Silico* prediction of differentially expressed miRNAs targeting epigenetic regulators

To evaluate miRNAs regulating epigenetic regulators and the potential for feedback loops within the sRNA biogenesis pathways we focused on the putative miRNA targets encoded within genes of the known epigenetic pathways described earlier (Yakovlev et al., 2016). All miRNAs and their predicted targets were analyzed, irrespective of their expression levels and distributing the target gene models by the type of epigenetic modification, they could be related to (Table 3, Table S9).

**Table 3:** Predicted miRNAs targeting epigenetic regulator with the distribution of the target gene models by the type of epigenetic modification.

	**Number of miRNAs**	**Number of target gene models**	**Number of DEMs**	**Number of target DEGs**
DNA methylation	4	5	3	3
Histone methylation	108	119	34	37
Histone acetylation	25	26	7	7
Histone (protein) phosphorylation	210	476	69	133
Histone ubiquitination (sumoylation)	24	29	9	9
Chromatin remodeling	17	17	7	7
sRNA pathways	21	16	4	4
Thermosensing	9	6	1	1
Total	364	683	120	197

In total, we found 84 conserved miRNAs from 32 families and 280 novel miRNAs that could target and post-transcriptionally regulate 683 gene models spanning all pathways of epigenetic regulation. Among them, 22 conserved miRNAs from 12 families and 98 novel miRNAs, with 197 of predicted targets, showed opposite transcription patterns and considered as involved into post-transcriptional silencing of epigenetic regulators during epitype formation in Norway spruce embryos (Table 3). As the miRNAs targets, we found several gene families, coding for genes involved in epigenetic regulation, like WD domain, G-beta repeat; SNF2 family N-terminal domain; DEAD/DEAH box helicase; BRCA1 C Terminus (BRCT) domain; KH (K Homology) RNA-binding domain; PHD-finger; Core histone H2A/H2B/H3/H4; SET domain; BTB/POZ (BR-C, ttk and bab/Pox virus and Zinc finger) domain; E1-E2 ATPase; ThiF family of Ubiquitin-activating enzyme (E1 enzyme) and some others.

We found only 4 miRNAs targeting the genes involved in DNA methylation, including methylation marks setting, erasing or reading (Table S9-1) and 3 miRNA-target pairs showed opposite transcription profiles, considering repression of target transcripts by high levels of miRNA transcripts and *vice versa*. 108 miRNAs were found to target 119 epigenetic regulators related to histone methylation (Table S9-2). Among them, 34 miRNA targeting 37 gene models, showed opposing transcriptional profiles. At least three SET-domain and one polycomb-like protein genes look to be regulated by conserved Pab-miR156.61 and three novel miRs—Pab-miRn0254.1_5p, Pab-miRn0305.2_3p, and Pab-miRn0252.1_3p correspondingly. In addition, large number of histone methylation readers could be regulated by miRNAs.

Phosphorylation was also an over-represented processes which appears to be highly regulated by miRNAs. Protein kinases (PK) were abundantly found among the gene families and had the highest number of total miRNA targets. We found 210 miRNAs, which could target 476 PK gene models, from which 69 miRNAs had opposite transcription profiles with 133 targets. Here we note, the conserved miRNAs from families' miR162, miR390, miR948, miR1313 and miR 3706 and abundant number of novel miRNAs, including Pab-miRn0117.1_3p; Pab-miRn0117.3_3p; Pab-miRn0165_3p; Pab-miRn0286.2_5p; Pab-miRn0301; Pab-miRn0367.1; Pab-miRn0441; Pab-miRn0592; Pab-miR1313.2 and others. All of them could target multiple gene models (Table S9-4).

Gene models related to histone acetylation were also revealed to be miRNA targets, but only 25 miRNAs were predicted to target such genes, and seven of these miRNA-target pairs had negatively correlated transcription profiles, indication miRNA regulatory effect (Table S9-3). Similarly, genes involved in ubiquitination/de-ubiquitination were infrequent amongst the miRNA targets. In this case, we found 24 miRNA targeting 29 gene models and 9 miRNA-target pairs with opposing transcriptional profiles (Table S9-5). Only 17 miRNAs were found to target chromatin remodelers, and these occurred mainly from the SNF2 family and in one case from a SWI gene family. In the latter case, 7 miRNA-target pairs had opposing transcription profiles, characteristic of a miRNA-target pair, and in each case these miRNAs were all of novel sequence composition (Table S9-6).

Conserved miRNAs were found to target genes participating in miRNA and other sRNAs biogenesis pathways. Several conserved miRNAs from miR156, miR162 and miR482 could target *DCL1-like* gene transcripts but transcription patterns did not confirm any regulatory effect. While, novels Pab-miRn miRn0030.3 shown to regulate *ARGONAUTE7* (*AGO7*), Pab-miRn0009.3_3p—3-hydroxy-3-methylglutaryl coenzyme A reductase (*HMG1*) and Pab-miRn0305.2_3p; Pab-miR482.29 shown to regulate different *SUPPRESSOR OF GENE SILENCING 3* (*SGS3*) genes (Table S9-7).

Another important and noteworthy observation was that a few genes were related to those already identified to be involved in thermosensing. Six genes were found to be potentially regulated by 9 miRNAs. We found just one pair Pab-miRn0407—Calcium-activated BK potassium channel (MA_10433576g0020) with opposite transcript profiles. Most of other genes belong to the Ankyrin repeat family and Myb-like DNA-binding domain, which could be targeted by numerous miRNAs, so it is difficult to establish clear transcription patterns using whole embryo samples (Table S9-8).

### Verification of miRNA-seq expressions by qRT-PCR

To validate the miRNA-Seq expression data, a subset of 10 specific DEMs (Table S1) was selected for verification by qRT-PCR. The selected miRNAs (5 conserved and 5 novel) showed a distinct differential profiles during embryo development at different epitype-inducing temperatures. For all of the 10 studied miRNAs the qRT-PCR outcomes closely correlated with the transcript abundance estimated by miRNA-Seq (Figure S4).

## Discussion

Different EpI temperatures of SE result in epigenetically different plants (epitypes) with altered bud phenology observed phenotypically later in the sporophytes annual growth cycle, in a predictable and reproducible manner (Kvaalen and Johnsen, 2008). During early development, complex gene expression, together with epigenetic changes, control and determine the embryonic cell fates (de Vega-Bartol et al., 2013; De-la-Peña et al., 2015). Some of these processes are determined by epigenetic modifications and networks of gene expression are directed and mediated by non-coding RNAs (Simon and Meyers, 2011; Lee, 2012; Mirouze, 2012). To focus on the genetic pathways initiating and establishing the epigenetic memory response, we obtained sRNAs using deep miRNA sequencing on embryos from contrasting EpI temperatures. Our sRNA analysis revealed over 3000 miRNA candidates from somatic embryonic tissues, including those of the 24-nt miRNA class. This result reconfirms their earlier found presence (Nystedt et al., 2013), in other gymnosperm species (Wan et al., 2012a,b; Wang et al., 2015), and firmly reject the earlier hypothesis that the 24 nt miRNA and siRNA class are missing and the notion that the DCL4-mediated miRNA biogenesis is absent within gymnosperm plants. We found a more complex structure of sRNA pool in embryonic tissues, compared to developed plant tissues, with the presence of additional longer fraction of sRNAs of 31–32 nt length. These sRNAs were not reported earlier in any plant species, but a same size class were described in animals germ line cells as PIWI-related sRNA (Le Thomas et al., 2014). Appearance of such longer fraction was very recently described during callus formation in tobacco plants Lunerova, (personal communication). Therefore, we could speculate that appearance of longer sRNA fraction could be related to pluripotent state of cells, presented in callus or developing SE. This longer sRNA fraction was not the topic of our present study and should be pursued further in a separate study. Aside from this, our *in silico analysis* uncovers numerous predicted novel miRNA families and identifies miRNAs with potential involvement in epigenetic regulation and thermosensing based on anti-correlated expression patterns of miRNA—target pairs.

Whilst this sRNA sequencing effort and *in silico* analysis is not an exhaustive search or analysis of the complex spruce sRNA population as we were limited only to one tissue type, it is the most exhaustive examination of 24–23 nt and 21–20 nt small RNAs in spruce to date. We limited our miRNA candidates' pool to predicted novel and conserved miRNAs with more than 10 reads in any libraries. In total, we identified 1115 miRNAs; nearly half of which (593) were putatively novel miRNAs and 522 conserved miRNAs. This conserved pool included 21 spruce specific miRNAs defined in previous studies (Yakovlev et al., 2010).

Analysis of the conserved miRNAs pool revealed a high number of miRNAs isoforms (isomiRs) nearly for all of the 58 conserved miRNA families. Various mechanisms are associated with the diversification of miRNA sequences, including imprecise DCL processing or post-transcriptional modifications, like trimming and tailing (Li et al., 2014). As we did not find specific genomic fragments with precursors for the most of isomiRs, most probably, they were originate from the common precursors and modified post-transcriptionally. Existence of isomiRs could be also explained by the possibility of miRNAs to originate from several MIRs. In this case, any SNP changes in miRNA genes would cause the creation of specific isomiRs. Generation of isomiRs from the same miRNA locus may extend its functional influence. As miRNA isoforms vary in size and sequence from the canonical miRNA, alternative targets may be regulated and differential AGO loading could arise, resulting in diverse regulatory outcomes (Neilsen et al., 2012; Ameres and Zamore, 2013; Li et al., 2014). Large amount of isoforms for conserved miRNAs are present in non-model species (e.g., Mica et al., 2009; Lin and Lai, 2013; Liu et al., 2014), but these isomiRs are not well characterized and, in most cases, their origins and functions if any remains unknown (Neilsen et al., 2012). It has previously been demonstrated that temperature treatments altered the expression of a specific subset of mature miRNAs and displayed differential expression of numbers of miRNA isoforms (Baev et al., 2014).

This is the first report of *miRNAs in gymnosperms. Even if our predictions detect the presence of both guide and *miRNA, we have not equivalently identified specific*miRNAs since such identification should be supported with more exact experimental knowledge of each miRNAs origin precursor. For most putative miRNA we found several potential precursors and we cannot at present define which exact precursor of origin or which arm of these precursors is the guiding (functional) mature miRNA and which is the passenger (non-functional) ones. We have defined miRNAs closer to 5′ end as -5p and putative ^*^miRNA on the opposite strand of the predicted precursor (closer to 3′ end) as -3p. In many cases both predicted miRNAs (guide and ^*^miRNA) from both strands were expressed and occasionally in opposite manner. Our sRNAs originate from whole embryos containing various tissues and cell types, and since different tissues and cell types may preferentially express either the -5p or the -3p variant (or vice versa), it is impossible in the present material to define with sufficient certainty which variant is main miRNA and which is star. Future cell type specific studies should be performed to clear up this matter.

Our target prediction revealed a large range of gene families with diverse biological functions. Usually, miRNAs regulate posttranslational repression of mRNAs via two different mechanisms: the miRNAs induce mRNA translational repression and the miRNAs induce mRNA cleavage under the help of ARGONAUTE protein (Bartel, 2009). Due to lack of necessity of full complementarity between miRNA and its target, it is difficult to establish clear relations between miRNA transcript profiles and their putative targets and transcript profiles as in many cases same gene transcript could be regulated by several miRNAs and, opposite, same miRNA could target several gene transcripts. High redundancy of miRNA—mRNA interplay and the multiplicity of miRNA genes and miRNA binding sites in the UTR of target genes may play a synergistic or additive function in the regulation of such targets (Bartel, 2009). Hence, the role of miRNA in gene expression would most likely be that of a fine-tuning process rather than an ON/OFF switch. One gene may be targeted by up and downregulated miRNA at the same time in order to attain the optimum concentration required for a specific function (Herranz and Cohen, 2010). This is the case for the mediation of cell fate decisions, where miRNAs act in synergy with other transcription regulators to establish gene regulatory networks (Herranz and Cohen, 2010).

We want to highlight the predictive nature of our *in silico* predictions using psRobot and psRNATarget analysis server and that future experimental validation studies are needed to confirm or refute our predictions. Notwithstanding, in most cases when we found inverse relations between miRNAs transcript levels and transcript levels of their predicted targets we presently considered them as a likely functional miRNA target pairs and have predicted their putative functional importance.

The highest number of predicted target gene models, which could be regulated by miRNAs, were found among multiple repeats containing proteins gene families, like: Leucine Rich Repeat (LRR) protein genes, protein kinase domain, NB-ARC (nucleotide-binding adaptor R-gene shared) domain, ATPase family associated with various cellular activities (AAA), Toll-Interleukin receptor (TIR) domain, Clathrin heavy chains/VPS (vacuolar protein sorting-associated), tetra- (TPR) and pentatricopeptide (PPR) repeat protein genes and some others. TIR-, NBS-LRR genes are considered as one of the first lines of defense against pathogen infection (Dangl and Jones, 2001; Meyers et al., 2005). However, our *in vitro* culture was axenic, free from any pathogens, and should not initiate any defense-related responses. Even so, we could see a large amount of differentially expressed LRR-containing genes showing clear dependence on epitype inducing temperature, and this imply their involvement in processes far removed from pathogen-mediated interactions. We consider that TIR, NBS, and LRR domain containing proteins may fulfill more general role in signals transduction from external environment (both biotic and abiotic) and conversion into molecular responses of diverse nature. TPR proteins can promote the formation of highly specific multiprotein complexes and can support the binding of different ligands (Zeytuni and Zarivach, 2012). A typical PPR protein could binds one or several organellar transcripts, and influences their expression by altering RNA sequence, turnover, processing, or translation (Barkan and Small, 2014). Clathrin and vacuolar protein sorting (VPS) domain proteins are another large group of protein involved in the vesicular sorting and trafficking pathways and essential for body plan development, defense and response to the environment (Chen et al., 2011). They often contain penta- and tetratricopeptide repeat domains, which could be the targets for regulation by miRNAs. In response to temperature differences, these may help fine tune intracellular traffic or the delivery of signaling molecules, but it is hard to envisage otherwise how they may directly contribute to temperature-dependent formation of epigenetic memory in the spruce embryos.

More specifically, we were looking for the genes involved in epigenetic regulation. It is shown, that a significant part of sRNA can serve as a pointer and participate in chromatin modification of promoters or DNA methylation, preventing, or activating the transcription of the individual is often remotely located sRNA coding genes or clusters (Mirouze, 2012). It is remarkably, that from the more than 700 gene models of putative epigenetic regulators described in developing embryos (Yakovlev et al., 2016), more than half are predicted targets by miRNAs. Moreover, we found that in EpI temperature dependent manner 197 DEGs of epigenetic regulators could be post-transcriptionally regulated by 120 miRNAs, including 22 conserved miRNAs from 12 families. miRNAs were mostly involved in regulation of genes related to methylation modifications, both in DNA and histones. In addition, several miRNAs were shown to target sRNA biogenesis pathway's gene models, confirming the existence of tight regulatory feedback loops within the miRNA and siRNA pathways in both gymnosperms and angiosperms (Henderson and Jacobsen, 2007; Niu et al., 2015). The opposite may occur, that miRNAs expression could be regulated by specific genes in response to changes in an extracellular microenvironment and considered as one of the major mechanisms for epigenetic modifications of the cell. It was shown that ion channels/transporters could transduce extracellular signals into miRNA transcript level changes, which, in turn, regulating target genes, and proposed potential link between cells and their microenvironment through ion channels/transporters (Jiang et al., 2012).

Most of miRNAs targeting epigenetic regulators predicted here were novel, so their possible existence also in other plant species should be pursued to verify their general importance. However, the fact that we find miRNA directly targeting all types of epigenetic modifiers indicated that miRNAs are central players involved in formation of epigenetic memory or at least in regulating the expression of the epigenetic machinery. In light of their important functions in the epigenetic memory formation, future validation work on these miRNAs and their targets is required.

## Conclusion

In this *in silico* analysis, we defined a predicted repertoire of conserved and novel miRNAs that could play crucial roles in regulating embryo development and epigenetic memory formation in Norway spruce. We showed that developing Norway spruce embryos possess a more complex sRNA structure than reported for somatic tissues. A variety of the predicted miRNAs showed distinct EpI temperature dependent expression patterns. These putative EpI miRNAs target spruce genes with a wide range of functions, including genes known to be involved in epigenetic regulation, which in turn could provide a feedback process leading to the formation of epigenetic marks. We suggest that TIR, NBS, and LRR domain containing proteins could fulfill more general functions for signal transduction from external environmental stimuli and conversion them into molecular response. Fine-tuning of the miRNA production likely participates in both developmental regulation and epigenetic memory formation in Norway spruce. This study also provides important information for comparative studies of miRNAs with other plant species and their predicted involvement in epigenetic regulation.

## Author contributions

IY and CF conceived and designed research. IY conducted experiments. IY analyzed data and wrote the manuscript together with CF. Both authors read and approved the final manuscript.

### Conflict of interest statement

The authors declare that the research was conducted in the absence of any commercial or financial relationships that could be construed as a potential conflict of interest.

## Abbreviations

DEG: differentially expressed gene
DEM: differentially expressed defined miRNA
SE: somatic embryogenesis
sRNA: small non-coding RNA
miRNA: microRNA
isomiRs: isoform microRNAs
EpI: epitype inducing.
