# Supplementary material for: In Silico Analysis of Small RNAs Suggest Roles for Novel and Conserved miRNAs in the Formation of Epigenetic Memory in Somatic Embryos of Norway Spruce
Source: Front Physiol. 2017 Sep 8;8:674. doi: 10.3389/fphys.2017.00674 (PMC5596105; doi:10.3389/fphys.2017.00674)
Supplement: Supplementary file 8 [file Table10.PDF]

Yakovlev et al. *In silico* analysis of small RNAs suggest roles for novel and conserved miRNAs in the formation of epigenetic memory in somatic embryos of Norway spruce

Supplement 10.

Table S10. Description of miRNA primers used for RT-PCR

##	miRNA ID	Forward primer (5′→ 3′)*	Primer length	Tm
1	Pab-miRn0076_5p	GGGCTTGGCAGAATCAGCGGG	21	70.5
2	Pab-miRn0081.1_5p	CCGAAAGTCGAGTTACGGTGC	21	62.2
3	Pab-miRn0082.3_5p	GGCTGTCGGCAGTCTGCCCGA	21	72.1
4	Pab-miRn0187	GGTCTTTCCACTTCTACCCATTTC	24 (22)	60.5
5	Pab-miRn0227.2_5p	TCCAACGAAGATCAGAAGGCTT	22	60.7
6	Pab-miR1316.3	GGATCTTCCATGCACAAACCATTA	24 (22)	63.0
7	Pab-miR167.1	GGAGATCATGTGGTAGCTTCAGC	23 (21)	60.6
8	Pab-miR319.4	TCTTGGACTGAAGGGAGCTCCCA	23	67.0
9	Pab-miR482.15	GTCTTCCCTACTCCTCCCATTCC	23	62.2
10	Pab-miR535.1	GTGACAACGAGAGAGAGCACGCA	23	65.1

* - mRQ 3’ Primer (Clontech) was used as reverse primer

Fig. S10. qRT-PCR validation for 10 differential expressed miRNAs, identified by miRNA-Seq in Norway spruce embryos developed under different epitype-inducing temperatures (18, 23 and 28°C). All data were averaged for 3 developmental stages, considered as biological replicates

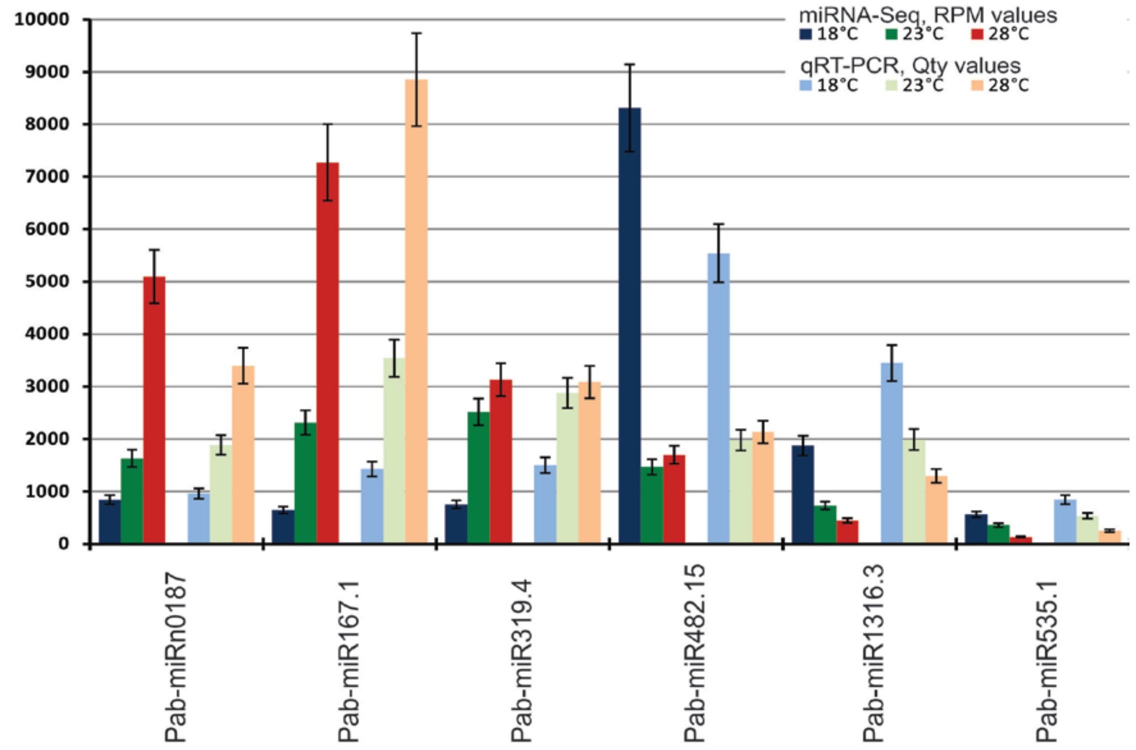
miRNA-Seq, RPM values
18°C
23°C
28°C
qRT-PCR, Qty values
18°C
23°C
28°C
10000
9000
8000
7000
6000
5000
4000
3000
2000
1000
0
Pab-miRn0187
Pab-miR167.1
Pab-miR319.4
Pab-miR482.15
Pab-miR1316.3
Pab-miR535.1

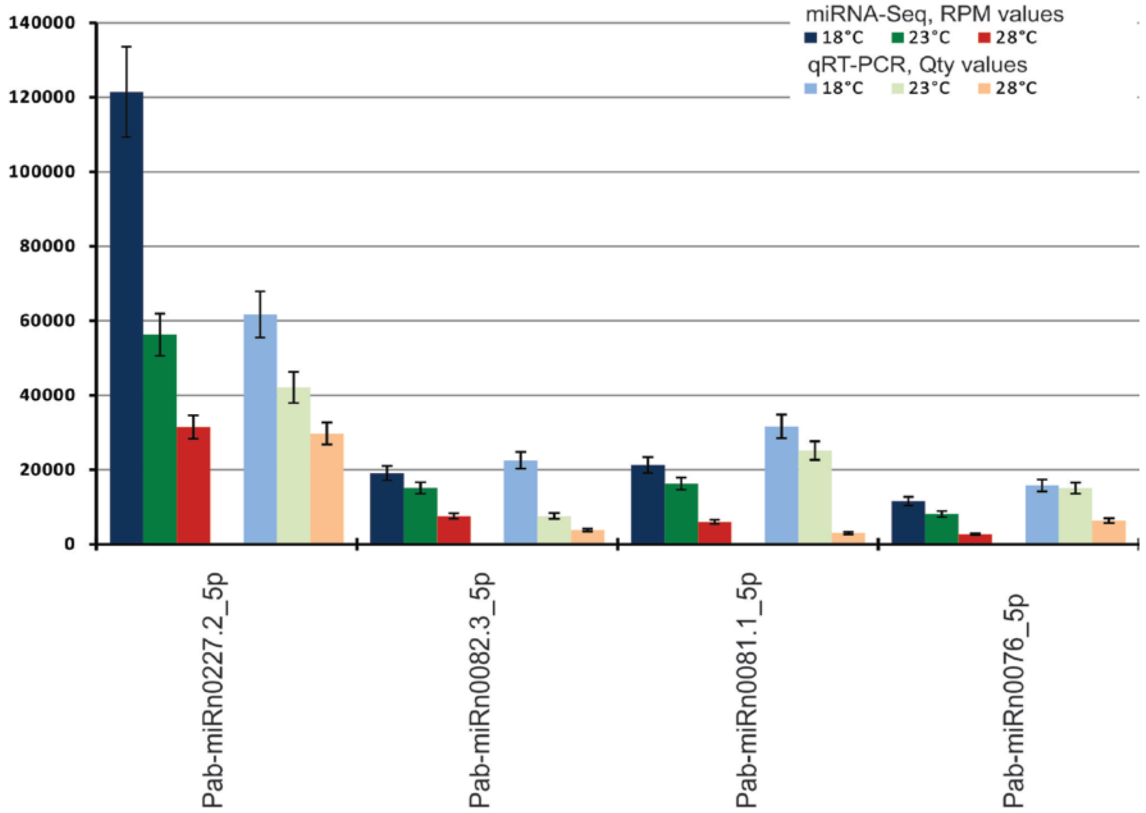
miRNA-Seq, RPM values
18°C
23°C
28°C
qRT-PCR, Qty values
18°C
23°C
28°C
140000
120000
100000
80000
60000
40000
20000
0
Pab-miRn0227.2_5p
Pab-miRn0082.3_5p
Pab-miRn0081.1_5p
Pab-miRn0076_5p
