# Supplementary material for: In Silico Analysis of Small RNAs Suggest Roles for Novel and Conserved miRNAs in the Formation of Epigenetic Memory in Somatic Embryos of Norway Spruce
Source: Front Physiol. 2017 Sep 8;8:674. doi: 10.3389/fphys.2017.00674 (PMC5596105; doi:10.3389/fphys.2017.00674)
Supplement: Supplementary file 9 [file TableS1andImageS1.PDF]

Yakovlev et al. *In silico* analysis of small RNAs suggest roles for novel and conserved miRNAs in the formation of epigenetic memory in somatic embryos of Norway spruce

Supplement 1.

General characterization of small RNAs libraries sequencings

Table S1. Characterization of sRNA libraries sequencing

Stage	Temperature °C	B10W		A2C	
		Number of reads	Number of reads 19-27 bp	Number of reads	Number of reads 19-27 bp
E1	18	1,779,786	562,695	3,613,553	972,560
E3	18	2,976,333	1,067,197	2,610,161	873,049
E5	18	1,883,502	599,129	2,968,265	1,042,090
E1	23	3,324,737	1,282,243	1,723,378	482,204
E3	23	2,132,285	912,569	3,972,804	1,558,642
E5	23	2,233,659	1,095,927	3,443,227	2,226,110
E1	28	2,200,882	948,667	3,962,137	2,392,329
E3	28	3,539,896	2,296,150	1,571,947	872,784
E5	28	2,143,487	1,564,086	3,576,153	2,262,926
Sum		22,214,567	10,328,663	27,441,625	12,682,694
Total A2C & B10W		49,656,192			23,011,357
Total A2C & B10W, more 2 reads					13,077,840

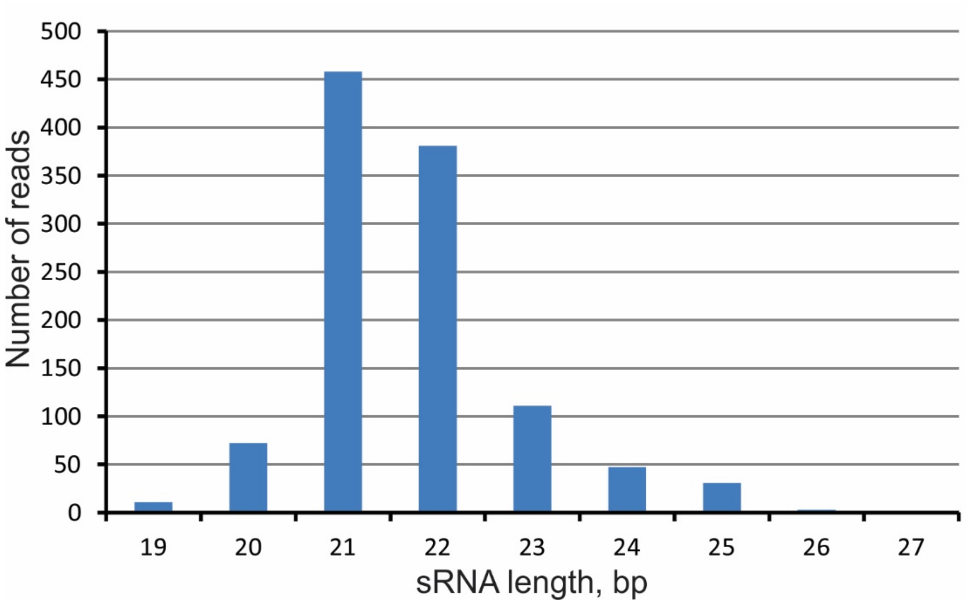

Figure S1. Length distribution in the *in silico* predicted defined novel and conserved miRNA in Norway spruce embryos
