# Supplementary material for: In Silico Analysis of Small RNAs Suggest Roles for Novel and Conserved miRNAs in the Formation of Epigenetic Memory in Somatic Embryos of Norway Spruce
Source: Front Physiol. 2017 Sep 8;8:674. doi: 10.3389/fphys.2017.00674 (PMC5596105; doi:10.3389/fphys.2017.00674)
Supplement: Supplementary file 10 [file Image2.PDF]

1a

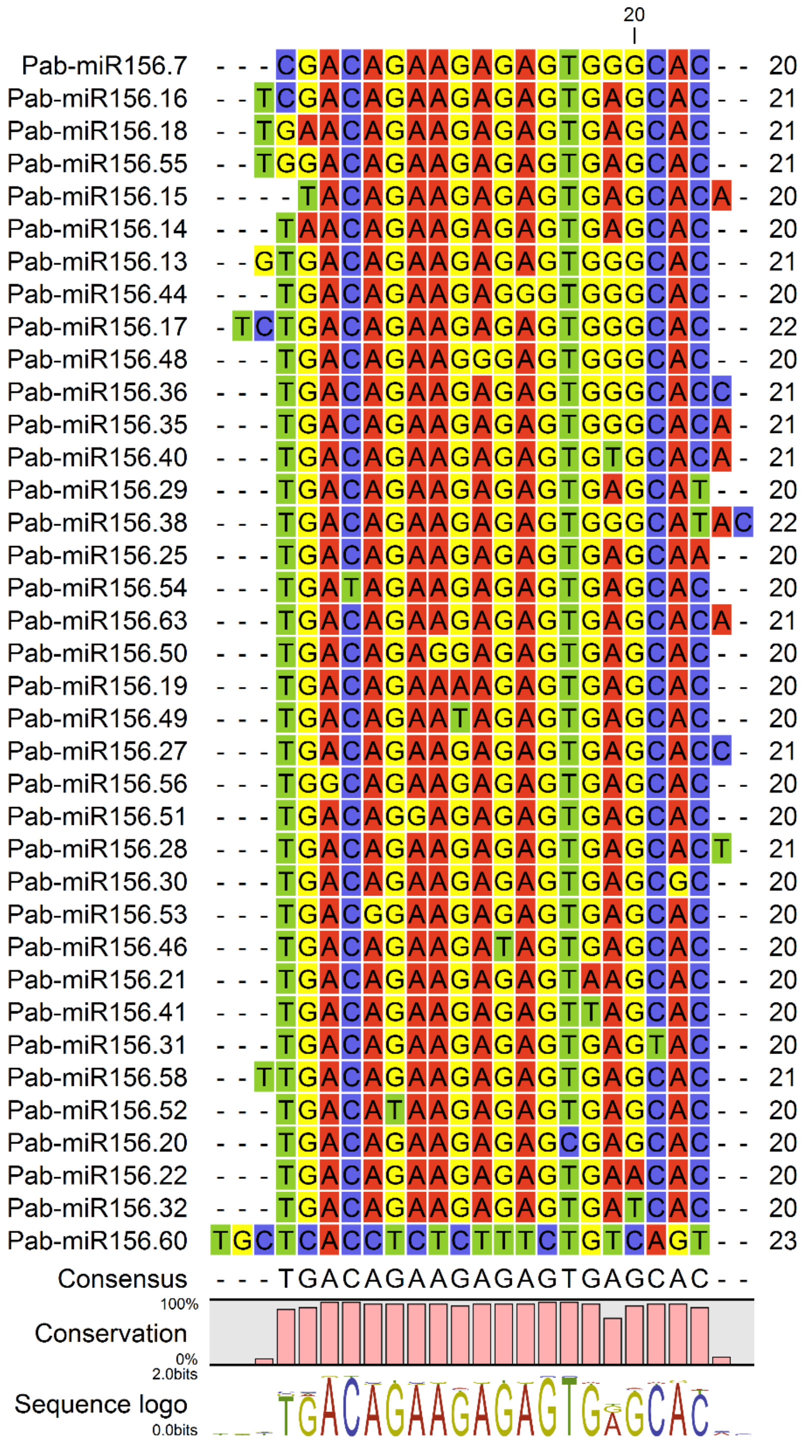
20
Pab-miR156.7 ---CGACAGAAGAGAGTGGCAC-- 20
Pab-miR156.16 --TCGACAGAAGAGAGTGAGCAC-- 21
Pab-miR156.18 --TGAACAGAAGAGAGTGAGCAC-- 21
Pab-miR156.55 --TGGACAGAAGAGAGTGAGCAC-- 21
Pab-miR156.15 ----TACAGAAGAGAGTGAGCACA- 20
Pab-miR156.14 ---TAACAGAAGAGAGTGAGCAC-- 20
Pab-miR156.13 --GTGACAGAAGAGAGTGGGCAC-- 21
Pab-miR156.44 ---TGACAGAAGAGGGTGGGCAC-- 20
Pab-miR156.17 -TCTGACAGAAGAGAGTGGGCAC-- 22
Pab-miR156.48 ---TGACAGAAGGGAGTGGGCAC-- 20
Pab-miR156.36 ---TGACAGAAGAGAGTGGGCACC- 21
Pab-miR156.35 ---TGACAGAAGAGAGTGGGCACA- 21
Pab-miR156.40 ---TGACAGAAGAGAGTGTGCACA- 21
Pab-miR156.29 ---TGACAGAAGAGAGTGAGCAT-- 20
Pab-miR156.38 ---TGACAGAAGAGAGTGGGCATAC 22
Pab-miR156.25 ---TGACAGAAGAGAGTGAGCAA-- 20
Pab-miR156.54 ---TGATAGAAGAGAGTGAGCAC-- 20
Pab-miR156.63 ---TGACAGAAGAGAGTGAGCACA- 21
Pab-miR156.50 ---TGACAGAGGAGAGTGAGCAC-- 20
Pab-miR156.19 ---TGACAGAAAAGAGTGAGCAC-- 20
Pab-miR156.49 ---TGACAGAATAGAGTGAGCAC-- 20
Pab-miR156.27 ---TGACAGAAGAGAGTGAGCACC- 21
Pab-miR156.56 ---TGGCAGAAGAGAGTGAGCAC-- 20
Pab-miR156.51 ---TGACAGGAGAGAGTGAGCAC-- 20
Pab-miR156.28 ---TGACAGAAGAGAGTGAGCACT- 21
Pab-miR156.30 ---TGACAGAAGAGAGTGAGCGC-- 20
Pab-miR156.53 ---TGACGGAAGAGAGTGAGCAC-- 20
Pab-miR156.46 ---TGACAGAAGATAGTGAGCAC-- 20
Pab-miR156.21 ---TGACAGAAGAGAGTAAGCAC-- 20
Pab-miR156.41 ---TGACAGAAGAGAGTTAGCAC-- 20
Pab-miR156.31 ---TGACAGAAGAGAGTGAGTAC-- 20
Pab-miR156.58 --TTGACAGAAGAGAGTGAGCAC-- 21
Pab-miR156.52 ---TGACATAAGAGAGTGAGCAC-- 20
Pab-miR156.20 ---TGACAGAAGAGAGCGAGCAC-- 20
Pab-miR156.22 ---TGACAGAAGAGAGTGAACAC-- 20
Pab-miR156.32 ---TGACAGAAGAGAGTGATCAC-- 20
Pab-miR156.60 TGCTCACCTCTCTTTCTGTCAGT-- 23
Consensus ---TGACAGAAGAGAGTGAGCAC--
100%
Conservation
0%
2.0bits
Sequence logo
0.0bits

1b

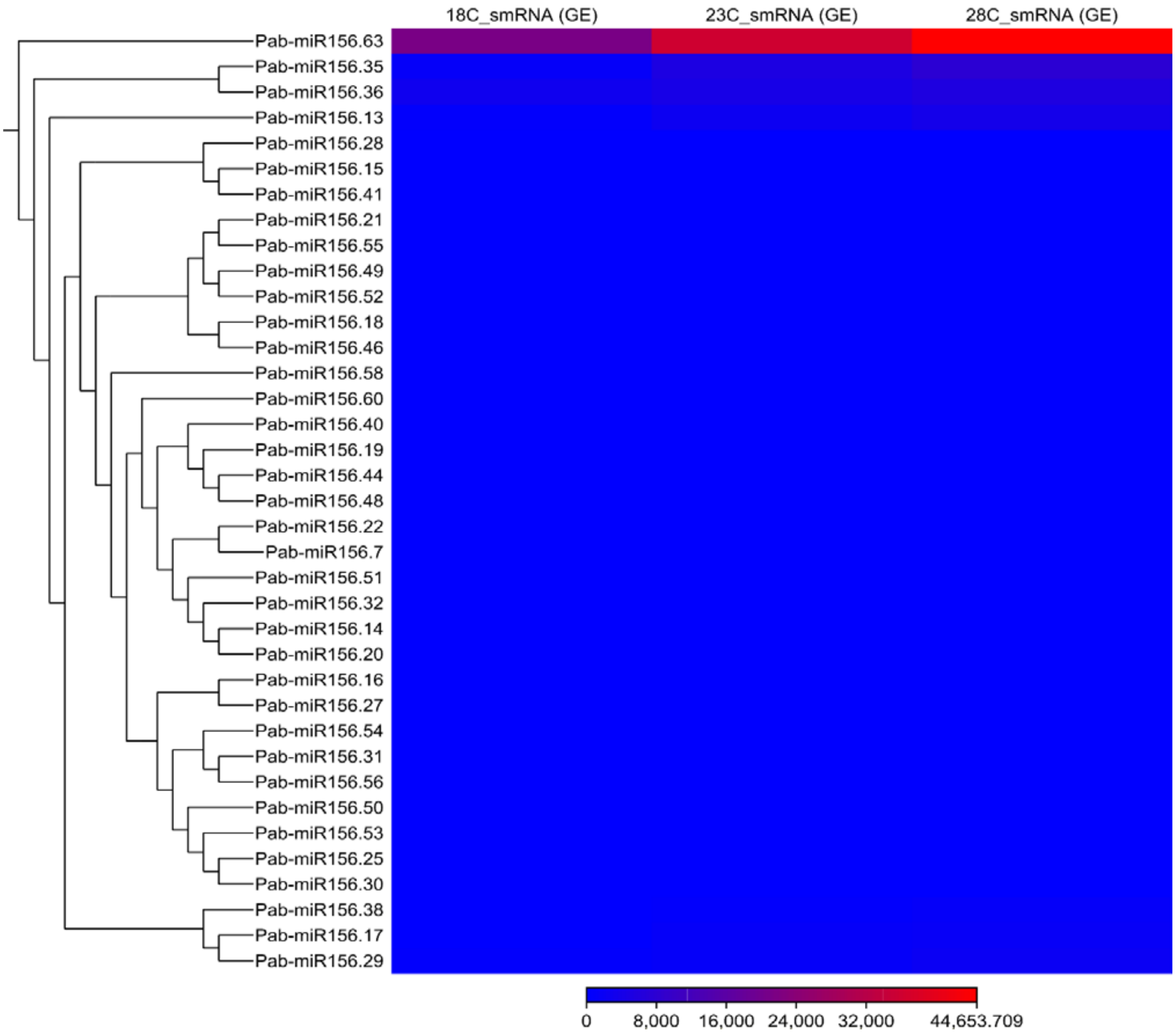

1c – Precursor MA_102488_prc1

UGAGUGCUGACAGAAGAGAGUGAGCACACAUAGCAGGAGCUCAUGAGACUAUGAGUUCAGCCAUGCGUGCUCACGUCUCUUUCUGUCAGCGGCUCA

(((((((((((((((((((((((((((.(((.((..(((((((((....))))))))).)).))).)))))))).)))).)))))))))).)))))

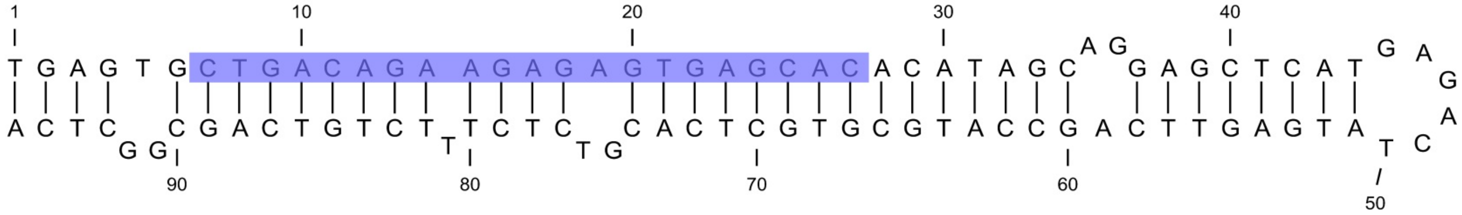

dG=-59.40 kcal/mol

2a

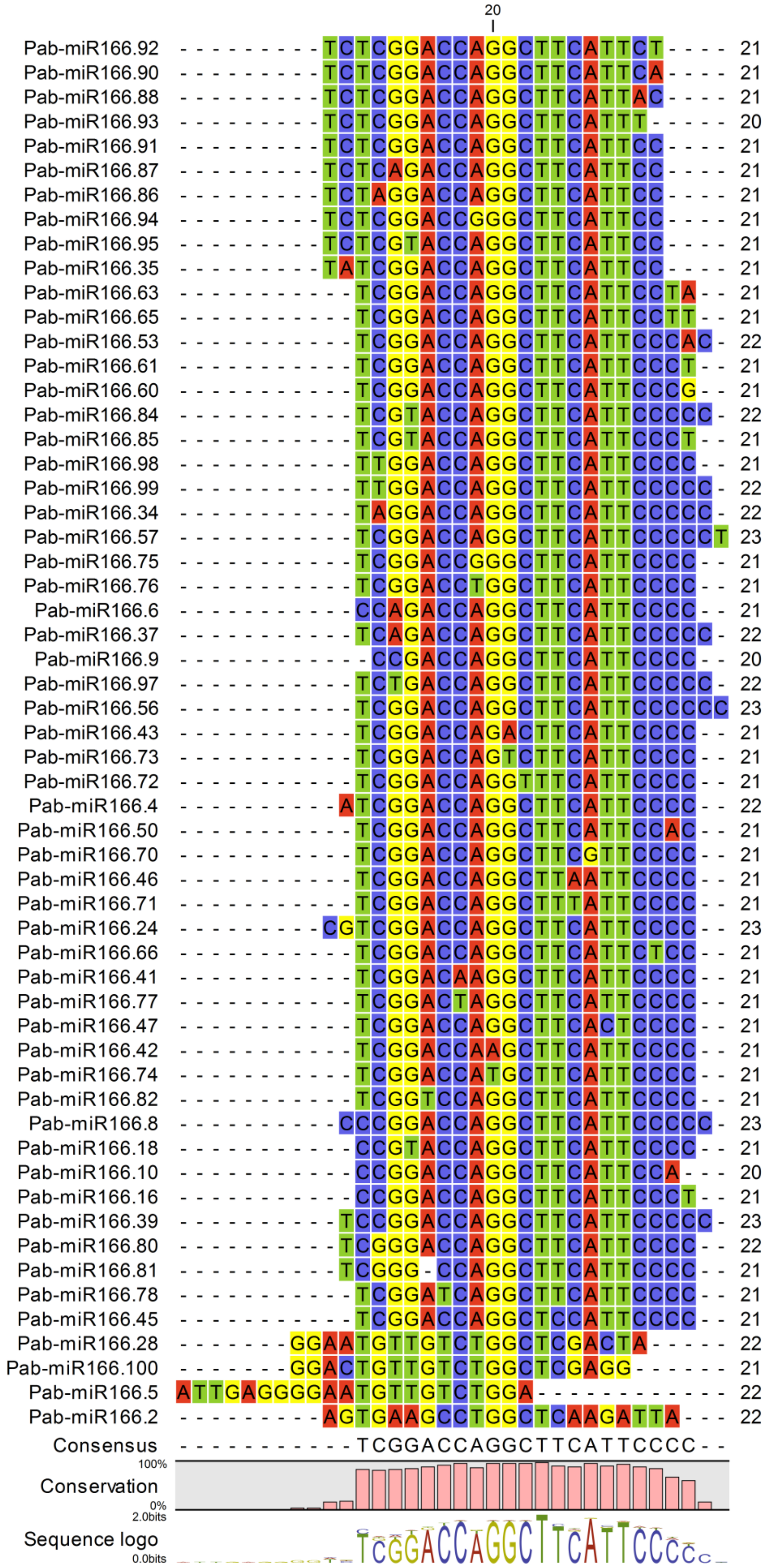
20
Pab-miR166.92 TCTCGGACCAGGCTTCATTCT 21
Pab-miR166.90 TCTCGGACCAGGCTTCATTCA 21
Pab-miR166.88 TCTCGGACCAGGCTTCATTAC 21
Pab-miR166.93 TCTCGGACCAGGCTTCATTT 20
Pab-miR166.91 TCTCGGACCAGGCTTCATTCC 21
Pab-miR166.87 TCTCAGACCAGGCTTCATTCC 21
Pab-miR166.86 TCTAGGACCAGGCTTCATTCC 21
Pab-miR166.94 TCTCGGACCGGGCTTCATTCC 21
Pab-miR166.95 TCTCGTACCAGGCTTCATTCC 21
Pab-miR166.35 TATCGGACCAGGCTTCATTCC 21
Pab-miR166.63 TCGGACCAGGCTTCATTCCTA 21
Pab-miR166.65 TCGGACCAGGCTTCATTCCTT 21
Pab-miR166.53 TCGGACCAGGCTTCATTCCCAC 22
Pab-miR166.61 TCGGACCAGGCTTCATTCCCT 21
Pab-miR166.60 TCGGACCAGGCTTCATTCCCG 21
Pab-miR166.84 TCGTACCAGGCTTCATTCCCCC 22
Pab-miR166.85 TCGTACCAGGCTTCATTCCCT 21
Pab-miR166.98 TTGGACCAGGCTTCATTCCCC 21
Pab-miR166.99 TTGGACCAGGCTTCATTCCCCC 22
Pab-miR166.34 TAGGACCAGGCTTCATTCCCCC 22
Pab-miR166.57 TCGGACCAGGCTTCATTCCCCCT 23
Pab-miR166.75 TCGGACCGGGCTTCATTCCCC 21
Pab-miR166.76 TCGGACCTGGCTTCATTCCCC 21
Pab-miR166.6 CCAGACCAGGCTTCATTCCCC 21
Pab-miR166.37 TCAGACCAGGCTTCATTCCCCC 22
Pab-miR166.9 CCGACCAGGCTTCATTCCCC 20
Pab-miR166.97 TCTGACCAGGCTTCATTCCCCC 22
Pab-miR166.56 TCGGACCAGGCTTCATTCCCCCC 23
Pab-miR166.43 TCGGACCAGACTTCATTCCCC 21
Pab-miR166.73 TCGGACCAGTCTTCATTCCCC 21
Pab-miR166.72 TCGGACCAGGTTTCATTCCCC 21
Pab-miR166.4 ATCGGACCAGGCTTCATTCCCC 22
Pab-miR166.50 TCGGACCAGGCTTCATTCCAC 21
Pab-miR166.70 TCGGACCAGGCTTCGTTCCCC 21
Pab-miR166.46 TCGGACCAGGCTTAATTCCCC 21
Pab-miR166.71 TCGGACCAGGCTTTATTCCCC 21
Pab-miR166.24 CGTCGGACCAGGCTTCATTCCCC 23
Pab-miR166.66 TCGGACCAGGCTTCATTCTCC 21
Pab-miR166.41 TCGGACAAGGCTTCATTCCCC 21
Pab-miR166.77 TCGGACTAGGCTTCATTCCCC 21
Pab-miR166.47 TCGGACCAGGCTTCACTCCCC 21
Pab-miR166.42 TCGGACCAAGCTTCATTCCCC 21
Pab-miR166.74 TCGGACCATGCTTCATTCCCC 21
Pab-miR166.82 TCGGTCCAGGCTTCATTCCCC 21
Pab-miR166.8 CCCGGACCAGGCTTCATTCCCCC 23
Pab-miR166.18 CCGTACCAGGCTTCATTCCCC 21
Pab-miR166.10 CCGGACCAGGCTTCATTCCA 20
Pab-miR166.16 CCGGACCAGGCTTCATTCCCT 21
Pab-miR166.39 TCCGGACCAGGCTTCATTCCCCC 23
Pab-miR166.80 TCGGGACCAGGCTTCATTCCCC 22
Pab-miR166.81 TCGGG-CCAGGCTTCATTCCCC 21
Pab-miR166.78 TCGGATCAGGCTTCATTCCCC 21
Pab-miR166.45 TCGGACCAGGCTCCATTCCCC 21
Pab-miR166.28 GGAATGTTGTCTGGCTCGACTA 22
Pab-miR166.100 GGACTGTTGTCTGGCTCGAGG 21
Pab-miR166.5 ATTGAGGGGAATGTTGTCTGGA 22
Pab-miR166.2 AGTGAAGCCTGGCTCAAGATTA 22
Consensus TCGGACCAGGCTTCATTCCCC
100%
Conservation
0%
2.0bits
Sequence logo
0.0bits

2b

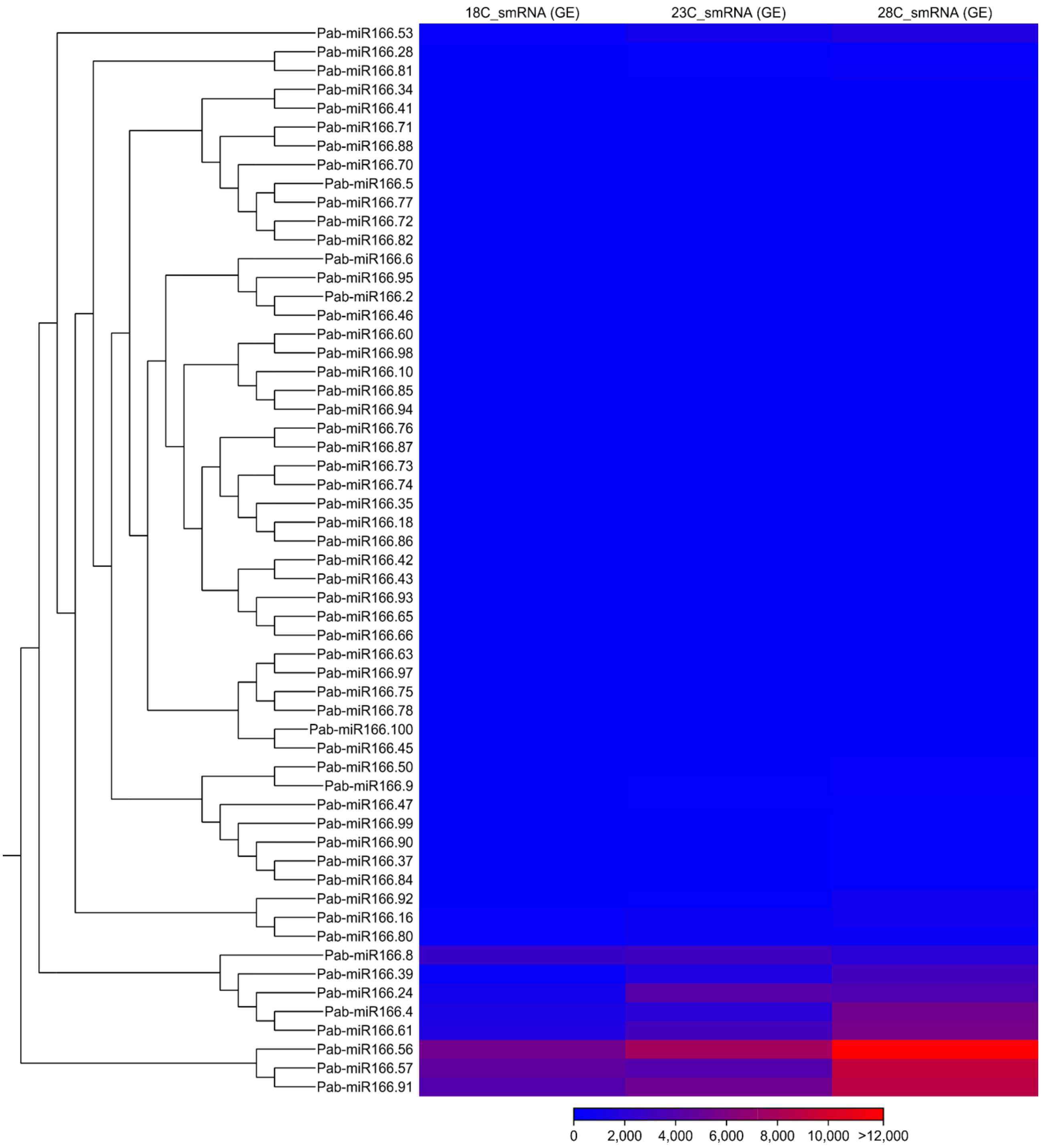
18C_smRNA (GE)
23C_smRNA (GE)
28C_smRNA (GE)
Pab-miR166.53
Pab-miR166.28
Pab-miR166.81
Pab-miR166.34
Pab-miR166.41
Pab-miR166.71
Pab-miR166.88
Pab-miR166.70
Pab-miR166.5
Pab-miR166.77
Pab-miR166.72
Pab-miR166.82
Pab-miR166.6
Pab-miR166.95
Pab-miR166.2
Pab-miR166.46
Pab-miR166.60
Pab-miR166.98
Pab-miR166.10
Pab-miR166.85
Pab-miR166.94
Pab-miR166.76
Pab-miR166.87
Pab-miR166.73
Pab-miR166.74
Pab-miR166.35
Pab-miR166.18
Pab-miR166.86
Pab-miR166.42
Pab-miR166.43
Pab-miR166.93
Pab-miR166.65
Pab-miR166.66
Pab-miR166.63
Pab-miR166.97
Pab-miR166.75
Pab-miR166.78
Pab-miR166.100
Pab-miR166.45
Pab-miR166.50
Pab-miR166.9
Pab-miR166.47
Pab-miR166.99
Pab-miR166.90
Pab-miR166.37
Pab-miR166.84
Pab-miR166.92
Pab-miR166.16
Pab-miR166.80
Pab-miR166.8
Pab-miR166.39
Pab-miR166.24
Pab-miR166.4
Pab-miR166.61
Pab-miR166.56
Pab-miR166.57
Pab-miR166.91
0
2,000
4,000
6,000
8,000
10,000
>12,000

2c – Precursor MA_10435792_prc1

UUGAGGGGAAUGUUGUCUGGCUCGAGGCCAUCCUGAAAAUAGUCCCCCGCCCCUUGGGGGUUUUCGGAUGCCGUCGGACCAGGCUUCAUUCCCCUCAA

(((((((((((..((((((.(((((((.((((.(((((.....((((((.....)))))))))))))))))).)))).))))))..))))))))))).

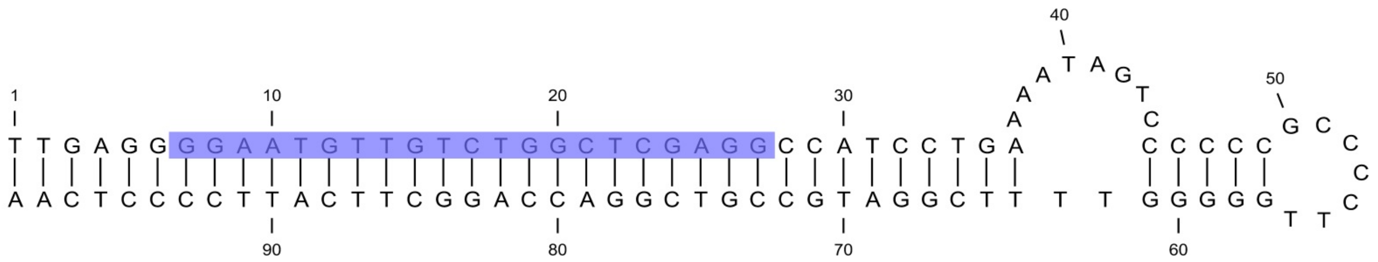

dG=-60.81 kcal/mol

3a

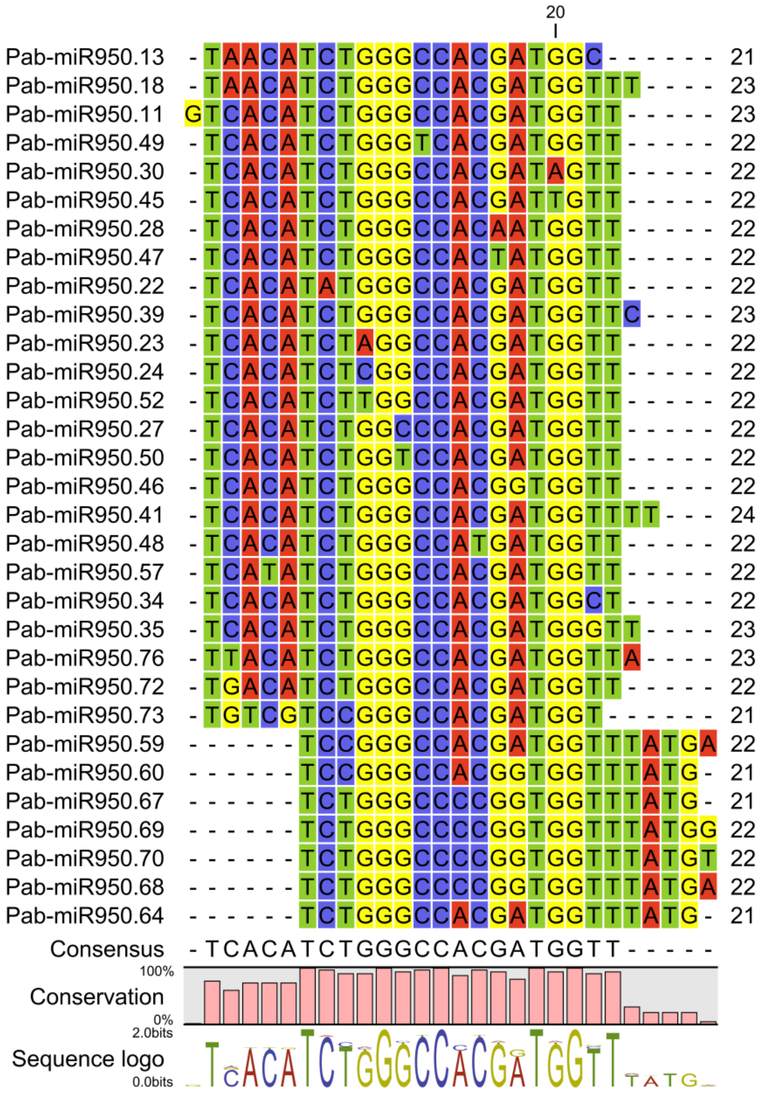
20
Pab-miR950.13 -TAACATCTGGGCCACGATGGC------ 21
Pab-miR950.18 -TAACATCTGGGCCACGATGGTTT---- 23
Pab-miR950.11 GTCACATCTGGGCCACGATGGTT----- 23
Pab-miR950.49 -TCACATCTGGGTCACGATGGTT----- 22
Pab-miR950.30 -TCACATCTGGGCCACGATAGTT----- 22
Pab-miR950.45 -TCACATCTGGGCCACGATTGTT----- 22
Pab-miR950.28 -TCACATCTGGGCCACAATGGTT----- 22
Pab-miR950.47 -TCACATCTGGGCCACTATGGTT----- 22
Pab-miR950.22 -TCACATATGGGCCACGATGGTT----- 22
Pab-miR950.39 -TCACATCTGGGCCACGATGGTTC---- 23
Pab-miR950.23 -TCACATCTAGGCCACGATGGTT----- 22
Pab-miR950.24 -TCACATCTCGGCCACGATGGTT----- 22
Pab-miR950.52 -TCACATCTTGGCCACGATGGTT----- 22
Pab-miR950.27 -TCACATCTGGCCCACGATGGTT----- 22
Pab-miR950.50 -TCACATCTGGTCCACGATGGTT----- 22
Pab-miR950.46 -TCACATCTGGGCCACGGTGGTT----- 22
Pab-miR950.41 -TCACATCTGGGCCACGATGGTTTT--- 24
Pab-miR950.48 -TCACATCTGGGCCATGATGGTT----- 22
Pab-miR950.57 -TCATATCTGGGCCACGATGGTT----- 22
Pab-miR950.34 -TCACATCTGGGCCACGATGGCT----- 22
Pab-miR950.35 -TCACATCTGGGCCACGATGGGTT---- 23
Pab-miR950.76 -TTACATCTGGGCCACGATGGTTA---- 23
Pab-miR950.72 -TGACATCTGGGCCACGATGGTT----- 22
Pab-miR950.73 -TGTCGTCCGGGCCACGATGGT------ 21
Pab-miR950.59 ------TCCGGGCCACGATGGTTTATGA 22
Pab-miR950.60 ------TCCGGGCCACGGTGGTTTATG- 21
Pab-miR950.67 ------TCTGGGCCCCGGTGGTTTATG- 21
Pab-miR950.69 ------TCTGGGCCCCGGTGGTTTATGG 22
Pab-miR950.70 ------TCTGGGCCCCGGTGGTTTATGT 22
Pab-miR950.68 ------TCTGGGCCCCGGTGGTTTATGA 22
Pab-miR950.64 ------TCTGGGCCACGATGGTTTATG- 21
Consensus -TCACATCTGGGCCACGATGGTT-----
100%
Conservation
0%
2.0bits
Sequence logo
0.0bits

3b

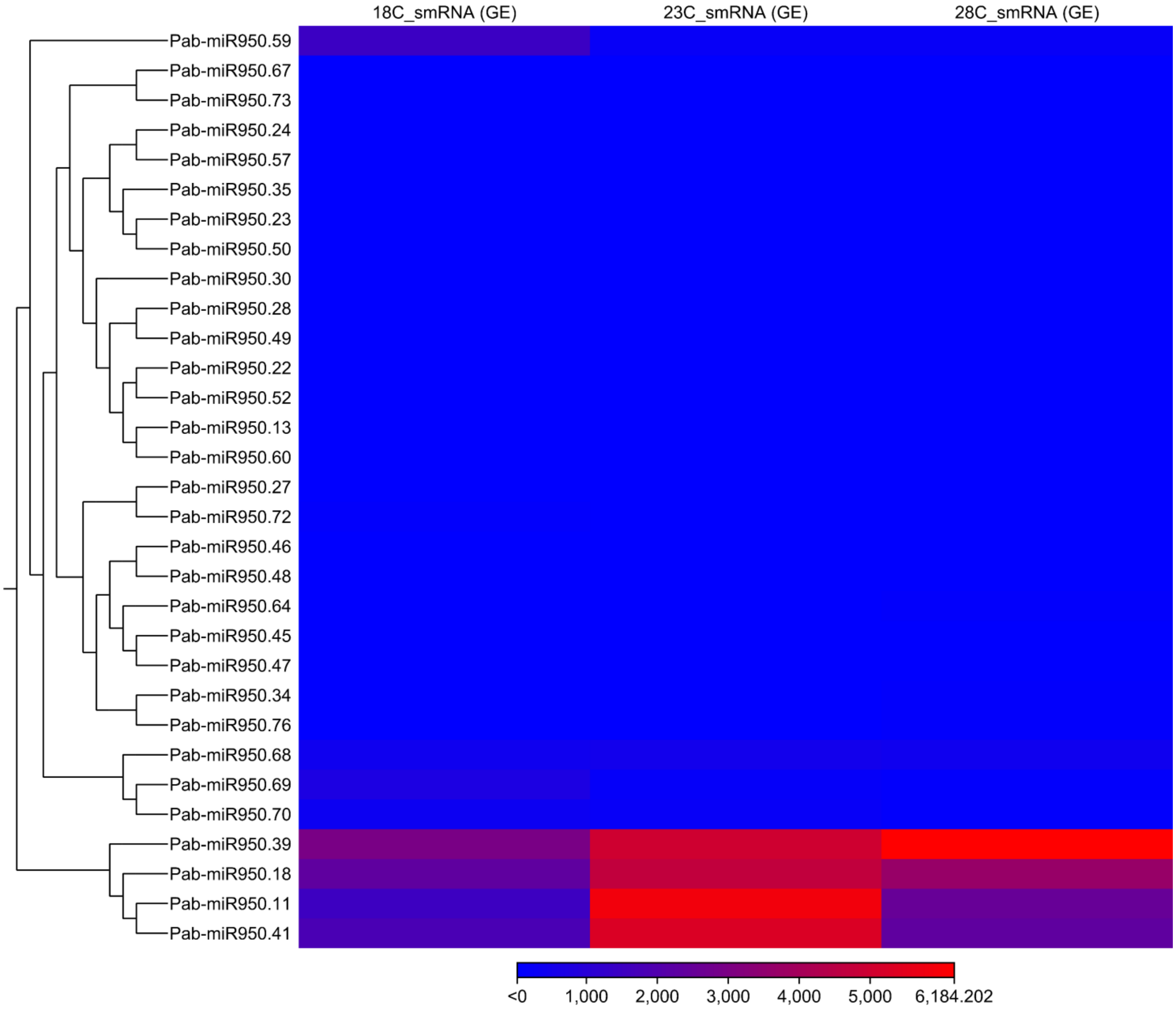

3c – Precursor MA_138737_prc1

UGUUAUUGUCGUCUGGGCCCCGGUGGUUUAUGAUCGUCUAGGAUGUUCAGAACCACCGUGGCCAAGACGACAAGAAUA
((((.(((((((((.((((.(((((((((.(((.((((...)))).)))))))))))).)))).))))))))).))))

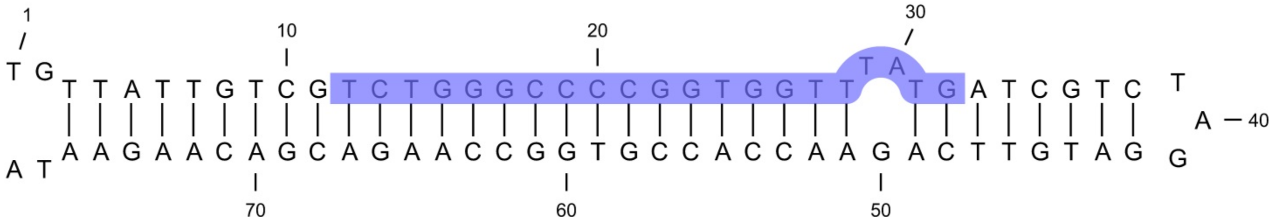

dG=-45.12 kcal/mol

Precursor MA_10432994_prc1

GAAAGUGUUCUUGACAUCUGGGCCACGAUGGUUUGUUUACCAUUUUAUGUCUAUAAACUAUCGUUGCCAGACGACACGAGCACCUUCG

(((.((((((.((...((((.((.((((((((((((..((........))..)))))))))))).))))))...)).)))))).))).

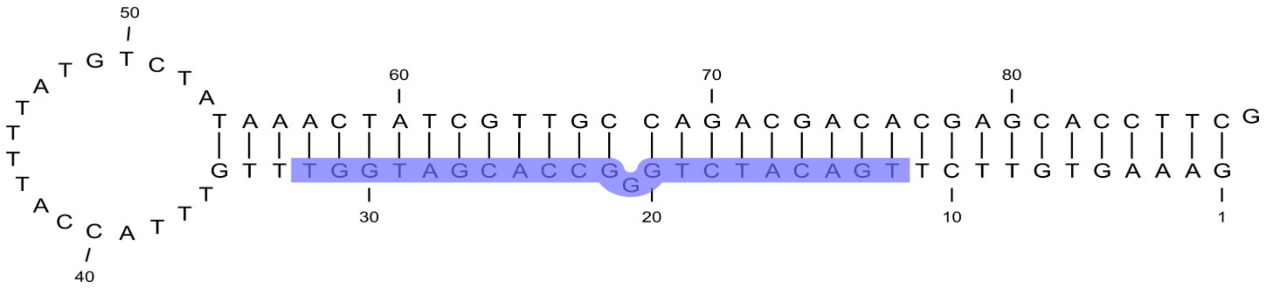

dG=-34.71 kcal/mol

4a

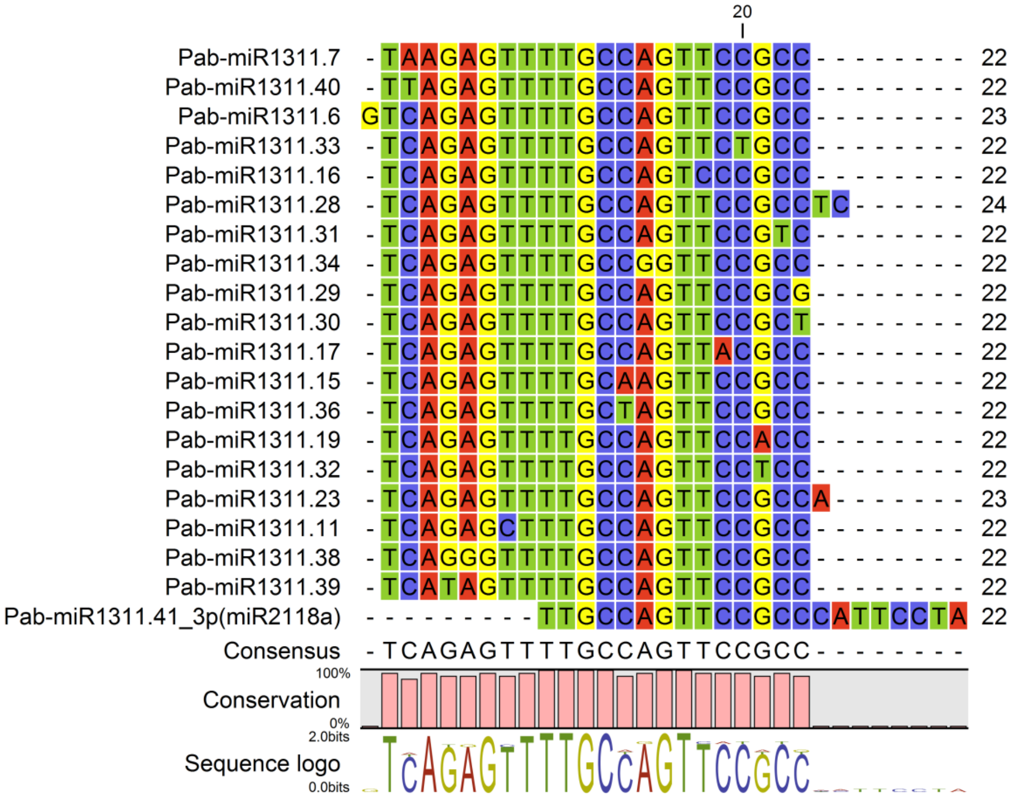
20
Pab-miR1311.7
Pab-miR1311.40
Pab-miR1311.6
Pab-miR1311.33
Pab-miR1311.16
Pab-miR1311.28
Pab-miR1311.31
Pab-miR1311.34
Pab-miR1311.29
Pab-miR1311.30
Pab-miR1311.17
Pab-miR1311.15
Pab-miR1311.36
Pab-miR1311.19
Pab-miR1311.32
Pab-miR1311.23
Pab-miR1311.11
Pab-miR1311.38
Pab-miR1311.39
Pab-miR1311.41_3p(miR2118a)
Consensus
-TCAGAGTTTTGCCAGTTCCGCC--------
100%
Conservation
0%
2.0bits
Sequence logo
0.0bits

4b

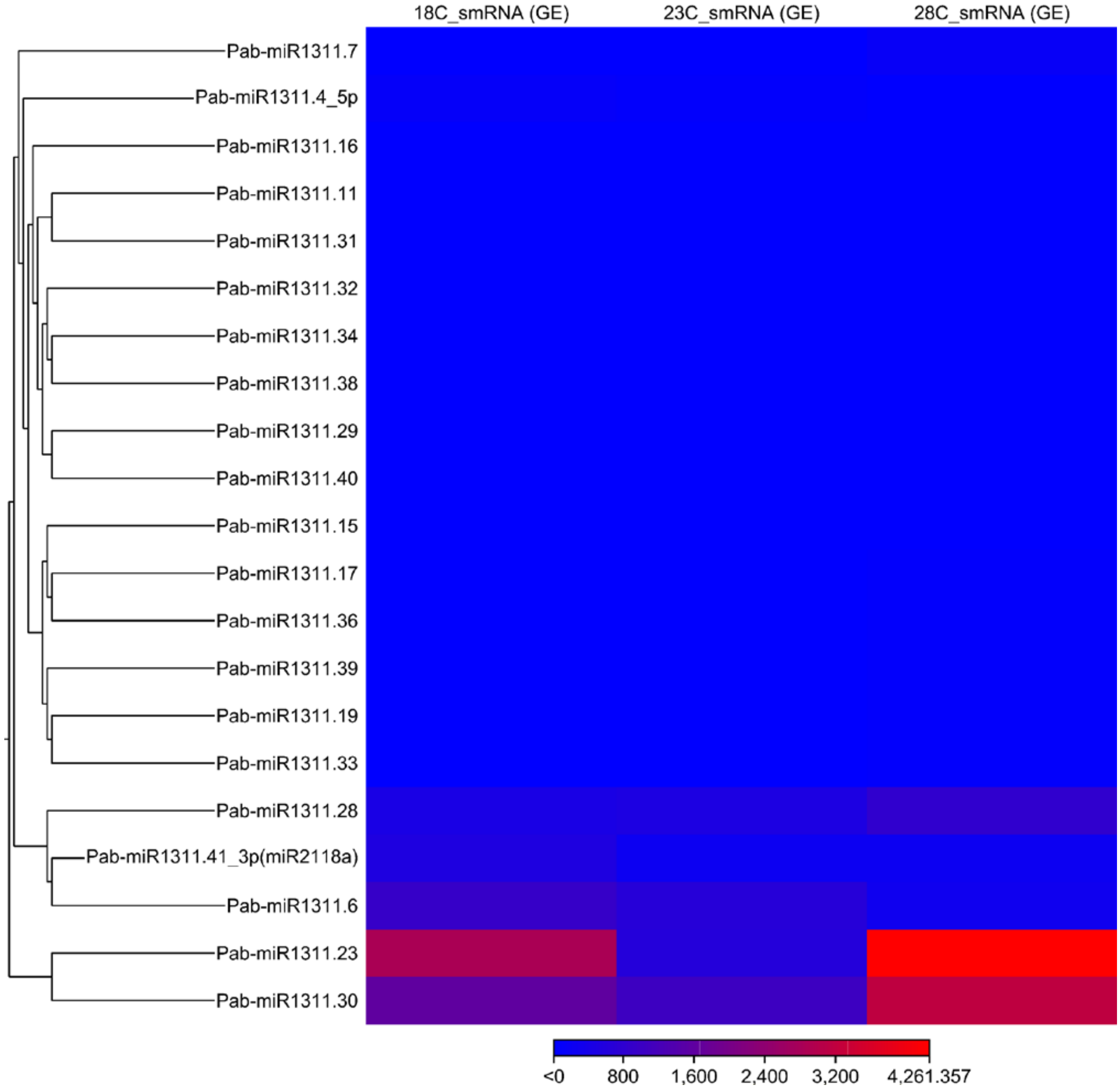
18C_smRNA (GE)
23C_smRNA (GE)
28C_smRNA (GE)
Pab-miR1311.7
Pab-miR1311.4_5p
Pab-miR1311.16
Pab-miR1311.11
Pab-miR1311.31
Pab-miR1311.32
Pab-miR1311.34
Pab-miR1311.38
Pab-miR1311.29
Pab-miR1311.40
Pab-miR1311.15
Pab-miR1311.17
Pab-miR1311.36
Pab-miR1311.39
Pab-miR1311.19
Pab-miR1311.33
Pab-miR1311.28
Pab-miR1311.41_3p(miR2118a)
Pab-miR1311.6
Pab-miR1311.23
Pab-miR1311.30
<0
800
1,600
2,400
3,200
4,261.357

4c – Precursor MA_138737_prc1

UAUGGGCUUGGUAGGAUUAGGCGCACUGGCAUAACUCUGACCAUAGUUUAUGGUCAGAGUUUUGCCAGUUCCGCCCAUUCCUAUUGUGCCUUUAA

...((((..(((((((...((((.(((((((.(((((((((((((...))))))))))))).))))))).))))...)))))))..))))....

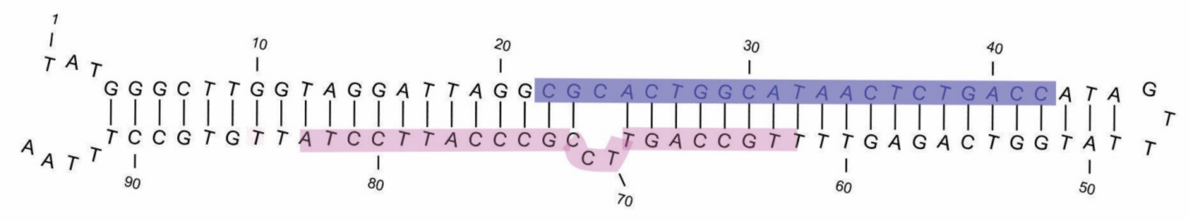

dG=-55.97 kcal/mol

Figure S2. Characterization of the differentially expressed members of the selected predicted conserved miRNA families in Norway spruce: 1 – Pab-miR156; 2 – Pab-miR166; 3 – Pab-miR950; 4 – Pab-miR1311; a – sequence alignment; b – Heatmap; c – precursor structure and folding model
