# Supplementary material for: In Silico Analysis of Small RNAs Suggest Roles for Novel and Conserved miRNAs in the Formation of Epigenetic Memory in Somatic Embryos of Norway Spruce
Source: Front Physiol. 2017 Sep 8;8:674. doi: 10.3389/fphys.2017.00674 (PMC5596105; doi:10.3389/fphys.2017.00674)
Supplement: Supplementary file 11 [file Image3.pdf]

Suppl. figure S3. Comparison of transcription profiles of selected differentially expressed predicted miRNA–gene functional pairs, involved in EpI temperature-dependent regulatory processes

##	Graph	Predicted target IDs	Pfam annotation
1.	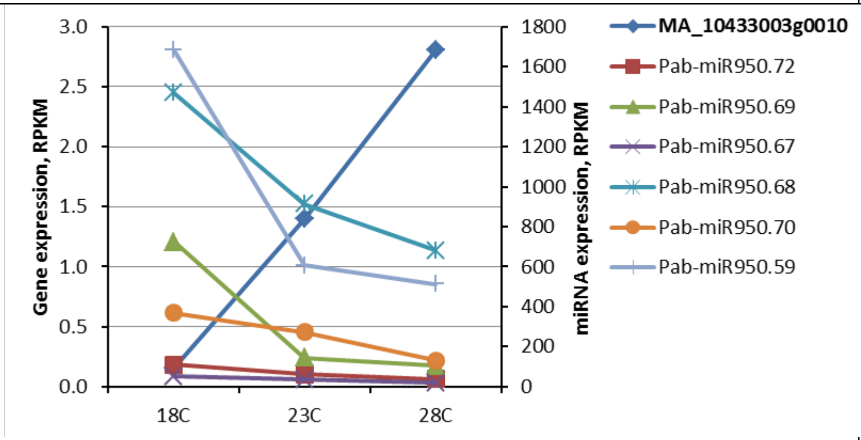	MA_10433003g0010	PF00931 - NB-ARC domain; PF01582; PF13676 - TIR domain; PF01637 - Archaeal ATPase; PF01656 - CobQ/CobB/MinD/ParA nucleotide binding domain; PF03205 - Molybdopterin guanine dinucleotide synthesis protein B; PF03308 - ArgK protein; PF04665 - Poxvirus A32 protein; PF05729 - NACHT domain; PF06745 - KaiC; PF08477 - Miro-like protein; PF13191 - AAA ATPase domain; PF13173; PF13207; ;PF13238; PF13401 - AAA domain
2.	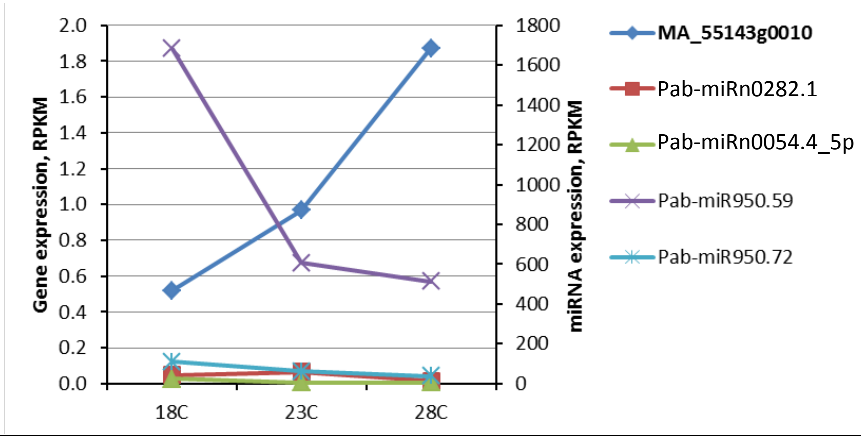	MA_55143g0010	PF00560 - Leucine Rich Repeat; PF00931 - NB-ARC domain;PF01582 - TIR domain; PF08937 - MTH538 TIR-like domain (DUF1863); PF12799 - Leucine Rich repeats (2 copies); PF12826 - Helix-hairpin-helix motif; PF13306 - Leucine rich repeats (6 copies);PF13504 - Leucine rich repeat; PF13676 - TIR domain; PF13855 - Leucine rich repeat

3.	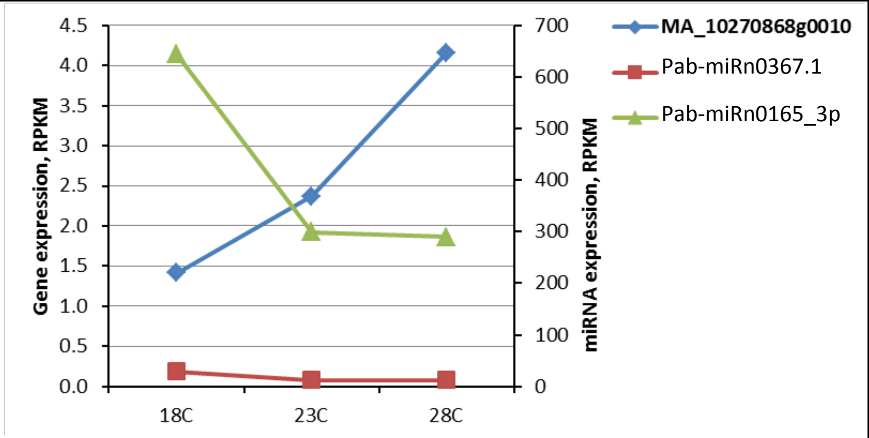	MA_10270868g0010	LRR receptor-like serine/threonine-protein kinase At3g47570 isoform X2 [Gossypium raimondii]
4.	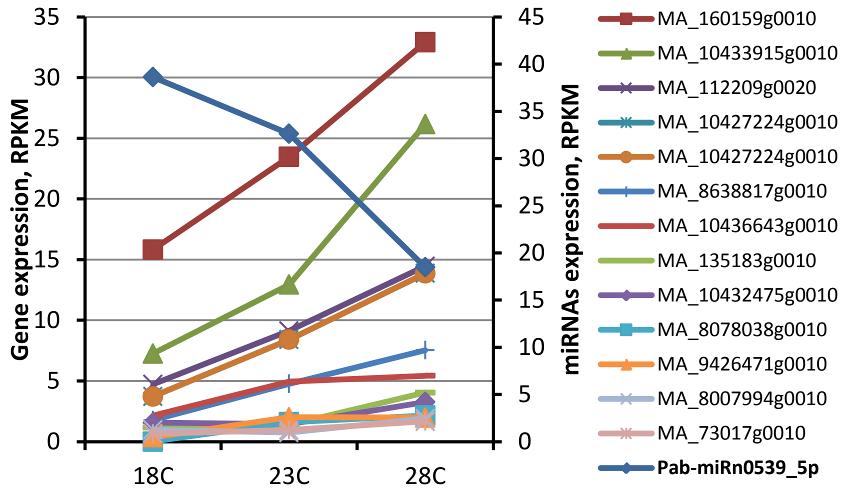	MA_8078038g0010<br>MA_9426471g0010<br>MA_8007994g0010<br>MA_10433915g0010<br><br>MA_10432475g0010<br>MA_10427224g0010<br><br>MA_112209g0020<br><br>MA_160159g0010<br><br>MA_8638817g0010<br><br>MA_73017g0010<br><br>MA_135183g0010<br><br>MA_10436643g0010	PF00931 - NB-ARC domain<br>PF12799 - Leucine Rich repeats (2 copies)<br>PF12799 - Leucine Rich repeats (2 copies)<br>PF00931 - NB-ARC domain; PF08561 - Mitochondrial ribosomal protein L37; PF11568 - Mediator complex subunit 29<br>PF00004 - ATPase family associated with various cellular activities (AAA); PF00931 - NB-ARC domain; PF01637 - Archaeal ATPase<br>PF00004 - ATPase family associated with various cellular activities (AAA); PF00931 - NB-ARC domain; PF01582 - TIR domain<br>PF00560 - Leucine Rich Repeat; PF00931 - NB-ARC domain; PF01582 - TIR domain<br>PF00004 - ATPase family associated with various cellular activities (AAA); PF00931 - NB-ARC domain; PF01637 - Archaeal ATPase<br>PF00004 - ATPase family associated with various cellular activities (AAA); PF00560 - Leucine Rich Repeat; PF00931 - NB-ARC domain<br>PF00560 - Leucine Rich Repeat; PF00931 - NB-ARC domain; PF01582 - TIR domain<br>PF00004 - ATPase family associated with various cellular activities (AAA); PF00025 - ADP-ribosylation factor family; PF00560 - Leucine Rich Repeat

5.	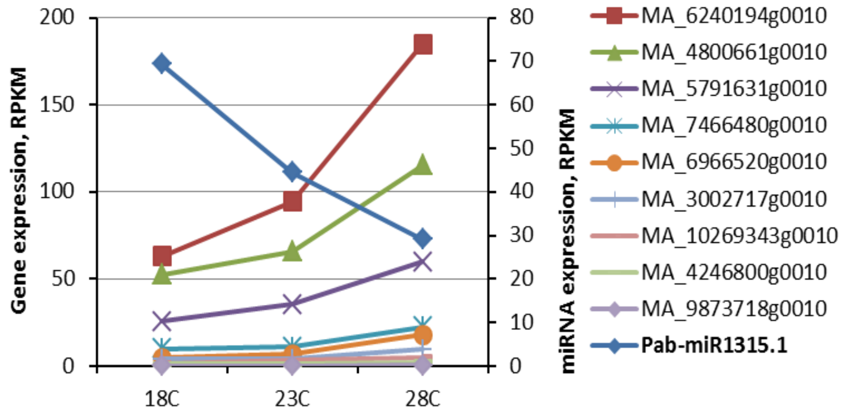	MA_10269343g0010<br>MA_4800661g0010<br>MA_5791631g0010<br>MA_9873718g0010<br>MA_3002717g0010<br>MA_6966520g0010<br>MA_7466480g0010<br>MA_6240194g0010<br>MA_4246800g0010	Unknown<br>Unknown<br>Unknown<br>Unknown<br>PF01715 - IPP transferase; PF10262 - Rdx family<br>PF06345 - DRF Autoregulatory Domain<br>PF06345 - DRF Autoregulatory Domain; PF10262 - Rdx family<br>PF10105 - Uncharacterized protein conserved in bacteria (DUF2344)<br>PF10262 - Rdx family
6.	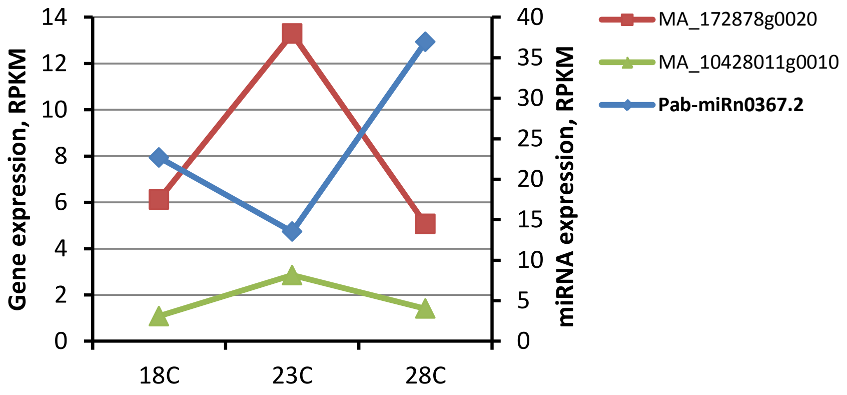	MA_172878g0020<br>MA_10428011g0010	PF00069 - Protein kinase domain; PF01636 - Phosphotransferase enzyme family; PF07714 - Protein tyrosine kinase<br>PF00069 - Protein kinase domain; PF00931 - NB-ARC domain; PF01582 - TIR domain; PF01636 - Phosphotransferase enzyme family; PF13191 - AAA ATPase domain
7.	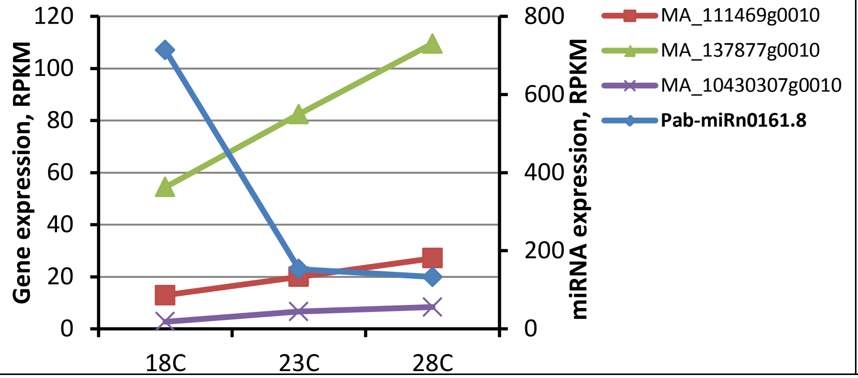	MA_111469g0010<br>MA_137877g0010<br>MA_10430307g0010	PF08879 - WRC (Trp, Arg, Cys) domain transcription factor; PF08880 - QLQ (Gln, Leu, Gln) motif at the N-terminus of SWI2/SNF2 protein<br>PF08879 - WRC (Trp, Arg, Cys) domain transcription factor ; PF08880 - QLQ (Gln, Leu, Gln) motif at the N-terminus of SWI2/SNF2 protein<br>PF00702 - haloacid dehalogenase-like hydrolase; PF12710 - haloacid dehalogenase-like hydrolase; PF13344 - Haloacid dehalogenase-like hydrolase

<table>
<tr><td>8.</td><td>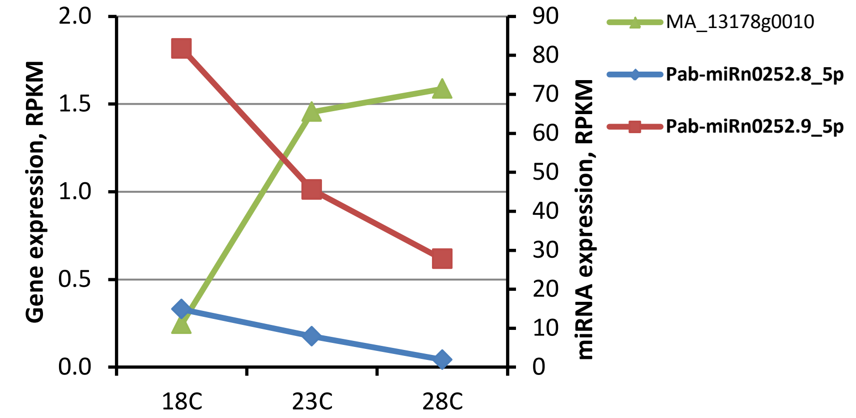
</td><td>MA_13178g0010</td><td>PF08167 - rRNA processing/ribosome biogenesis; PF11483 - Protein of unknown function (DUF3209); PF11865 - Domain of unknown function (DUF3385); PF13513 - HEAT-like repeat; PF13646 - HEAT repeats</td></tr>
<tr><td>9.</td><td>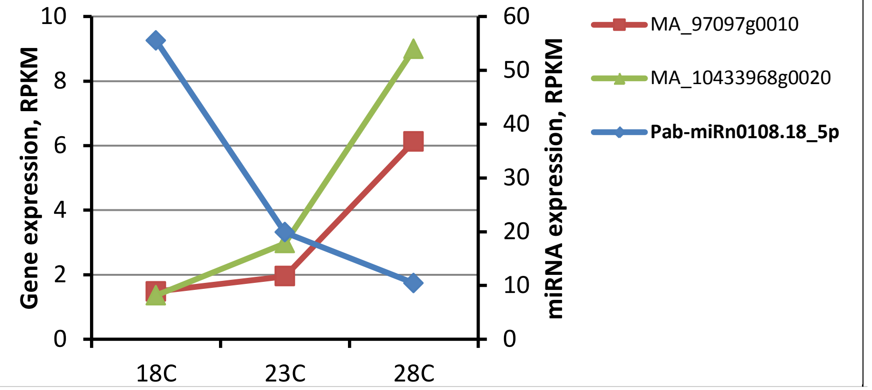
</td><td>MA_97097g0010

MA_10433968g0020</td><td>PF00097 - Zinc finger, C3HC4 type (RING finger); PF03031 - NLI interacting factor-like phosphatase; PF11793 - FANCL C-terminal domain; PF12678 - RING-H2 zinc finger; PF12861 - Anaphase-promoting complex subunit 11 RING-H2 finger
PF03031 - NLI interacting factor-like phosphatase; PF07527 - Hairy Orange domain</td></tr>
<tr><td>10.</td><td>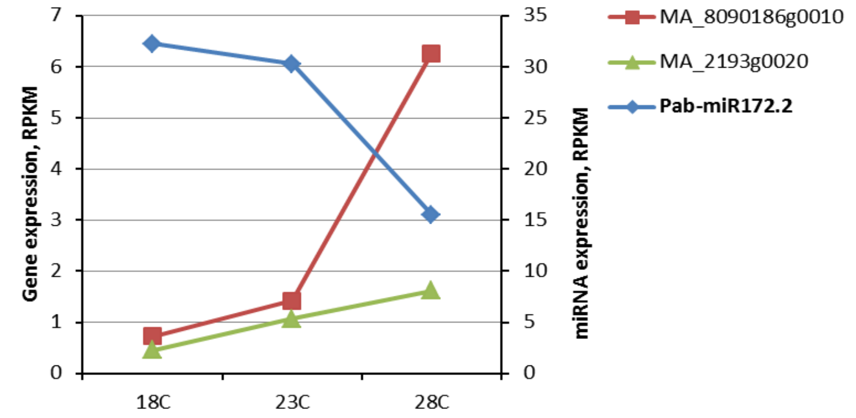
</td><td>MA_8090186g0010
MA_2193g0020</td><td>PF00847 - AP2 domain
PF00847 - AP2 domain</td></tr>
</table>

<table>
<tr><td>11.</td><td>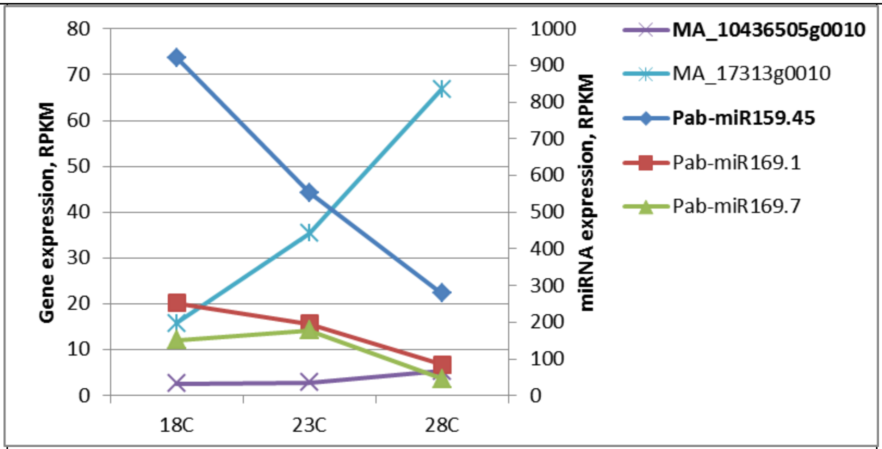
</td><td>MA_10436505g0010
MA_17313g0010</td><td>PF08744 - Plant transcription factor NOZZLE
PF02045 - CCAAT-binding transcription factor (CBF-B/NF-YA) subunit B</td></tr>
<tr><td>12.</td><td>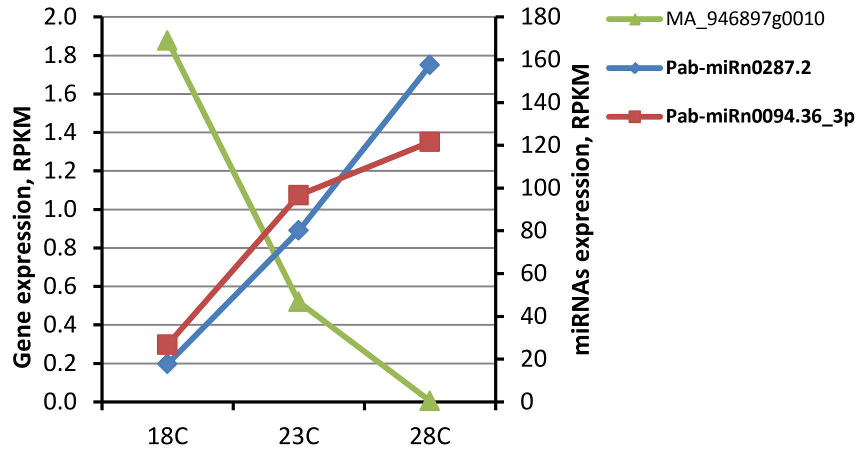
</td><td>MA_946897g0010</td><td>PF08506 - N-terminal Cse1 (chromosome-segregation protein) domain protein</td></tr>
</table>

13.	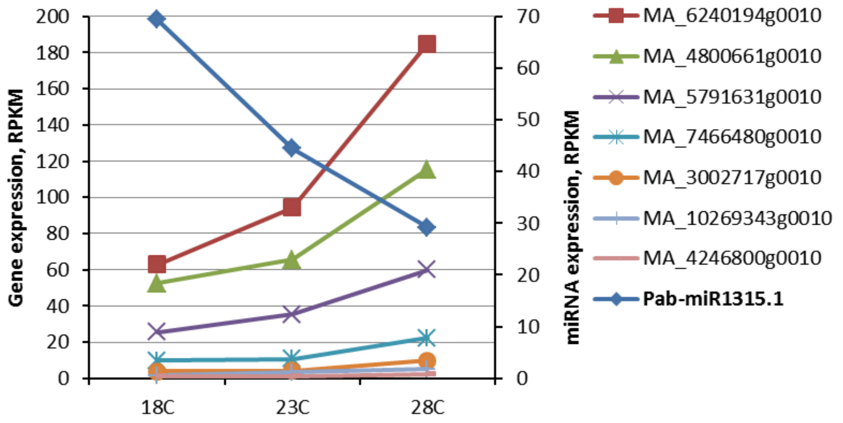	MA_6240194g0010	PF10105 - Uncharacterized protein conserved in bacteria (DUF2344)
		MA_4800661g0010	no match
		MA_5791631g0010	no match
		MA_7466480g0010	PF06345 - DRF Auto-regulatory Domain; PF10262 - Rdx family of selenoproteins
		MA_3002717g0010	PF01715 - tRNA Delta(2)-isopentenylpyrophosphate transferase (IPP transferase); PF10262 - Rdx family of selenoproteins
		MA_10269343g0010	no match
		MA_4246800g0010	no match
14.	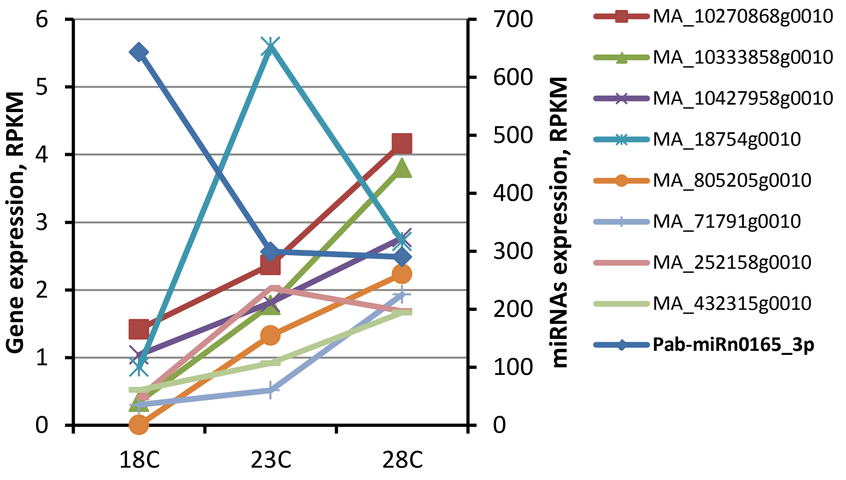	MA_10270868g0010	No match
		MA_10333858g0010	No match
		MA_10427958g0010	PF00069 - Protein kinase domain; PF00560 - Leucine Rich Repeat; PF01636 - Phosphotransferase enzyme family; PF03109 - ABC1 family; PF03116 - NQR2, RnfD, RnfE family; PF07714 - Protein tyrosine kinase; PF08263 - Leucine rich repeat N-terminal domain; PF12799 - Leucine Rich repeats (2 copies)
		MA_18754g0010	PF07887 - Calmodulin binding protein-like
		MA_805205g0010	No match
		MA_71791g0010	No match
		MA_252158g0010	No match
		MA_432315g0010	PF00069 - Protein kinase domain; PF00560 - Leucine Rich Repeat; PF07714 - Protein tyrosine kinase; PF12799 - Leucine Rich repeats (2 copies)

15.	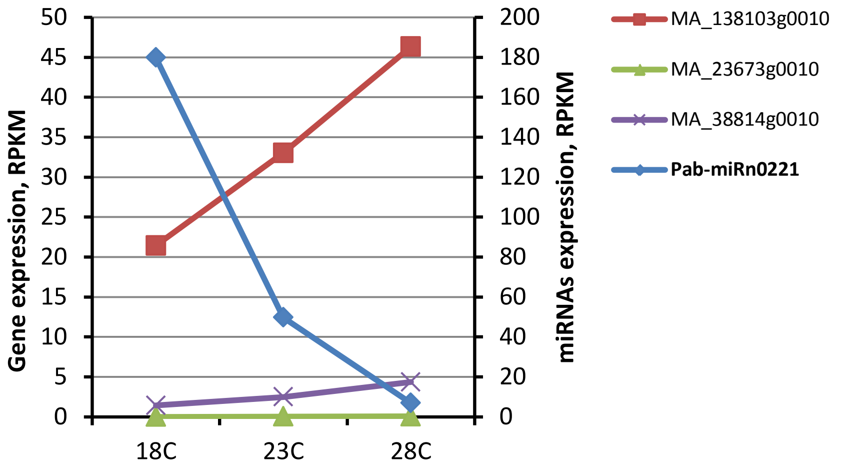	MA_138103g0010<br>MA_23673g0010<br>MA_38814g0010	PF00646 - F-box domain; PF12937 - F-box-like<br>PF00847 - AP2 domain; PF02362 - B3 DNA binding domain<br>PF00646 - F-box domain; PF01344 - Kelch motif; PF07646 - Kelch motif; PF12937 - F-box-like; PF13964 - Kelch motif
16.	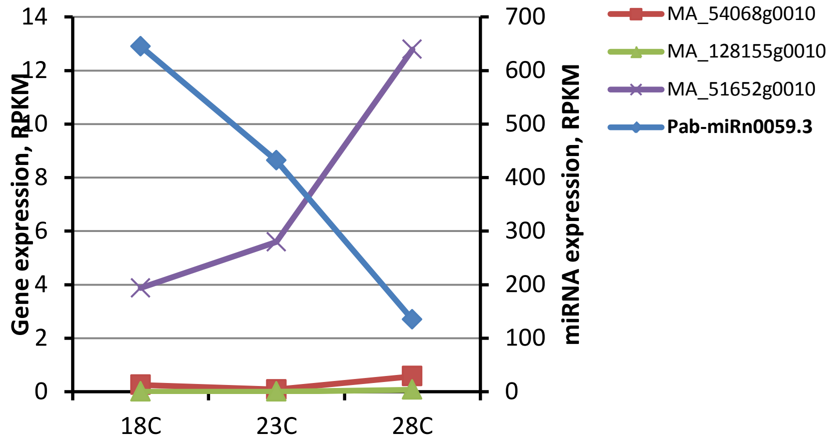	MA_54068g0010<br><br>MA_128155g0010<br>MA_51652g0010	PF00420 - NADH-ubiquinone/plastoquinone oxidoreductase chain 4L<br>No match<br>PF08744 - Plant transcription factor NOZZLE

17.	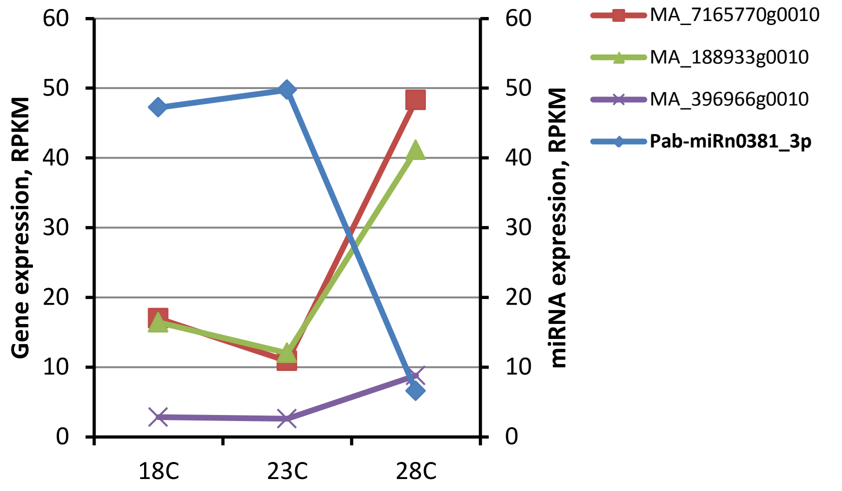	MA_7165770g0010	PF01397 - Terpene synthase, N-terminal domain; PF13249 - Prenyltransferase-like
		MA_188933g0010	PF01397 - Terpene synthase, N-terminal domain
		MA_396966g0010	PF01397 - Terpene synthase, N-terminal domain; PF13249 - Prenyltransferase-like
18.	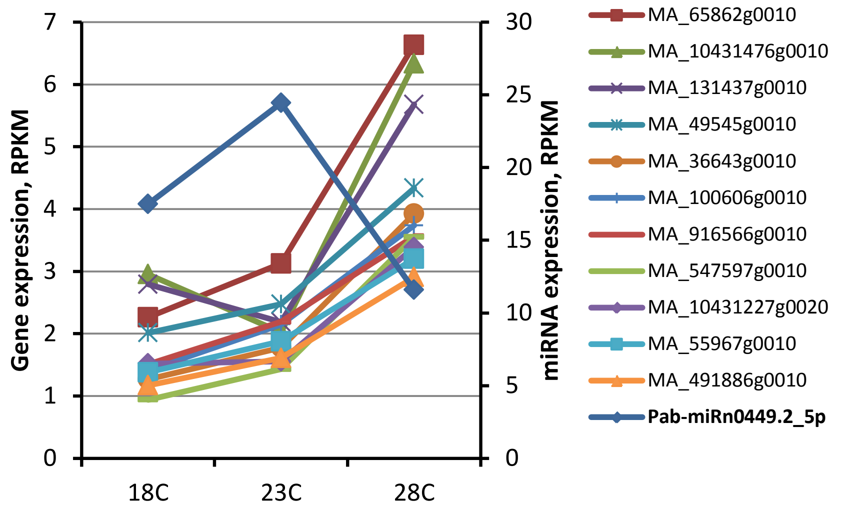	MA_65862g0010	PF00637 - Region in Clathrin and VPS; PF01535 - PPR repeat; PF04733 - Coatomer epsilon subunit
		MA_10431476g0010	PF01535 - PPR repeat; PF12854 - PPR repeat; PF13041 - PPR repeat family ; PF13431 - Tetratricopeptide repeat
		MA_131437g0010	
		MA_49545g0010	PF01535 - PPR repeat; PF12854 - PPR repeat; PF13041 - PPR repeat family ; PF13812 - Pentatricopeptide repeat domain
		MA_36643g0010	PF01535 - PPR repeat; PF03704 - Bacterial transcriptional activator domain; PF10602 - 26S proteasome subunit RPN7; PF12854 - PPR repeat
		MA_100606g0010	PF00418 - Tau and MAP protein, tubulin-binding repeat; PF00515 - Tetratricopeptide repeat; PF00637 - Region in Clathrin and VPS; PF01535 - PPR repeat
		MA_916566g0010	PF00515 - Tetratricopeptide repeat; PF01535 - PPR repeat; PF02758 - PAAD/DAPIN/Pyrin domain; PF04733 - Coatomer epsilon subunit
		MA_547597g0010	PF01535 - PPR repeat; PF03704 - Bacterial transcriptional activator domain; PF07443 - HepA-related protein (HARP); PF07719 - Tetratricopeptide repeat
		MA_10431227g0020	PF00515 - Tetratricopeptide repeat; PF01535 - PPR repeat; PF03704 - Bacterial transcriptional activator domain
		MA_55967g0010	PF00515 - Tetratricopeptide repeat; PF01535 - PPR repeat;

			PF03704 - Bacterial transcriptional activator domain; PF07719 - Tetratricopeptide repeat
		MA_491886g0010	PF01535 - PPR repeat; PF10602 - 26S proteasome subunit RPN7; PF12854 - PPR repeat; PF13041 - PPR repeat family
19.	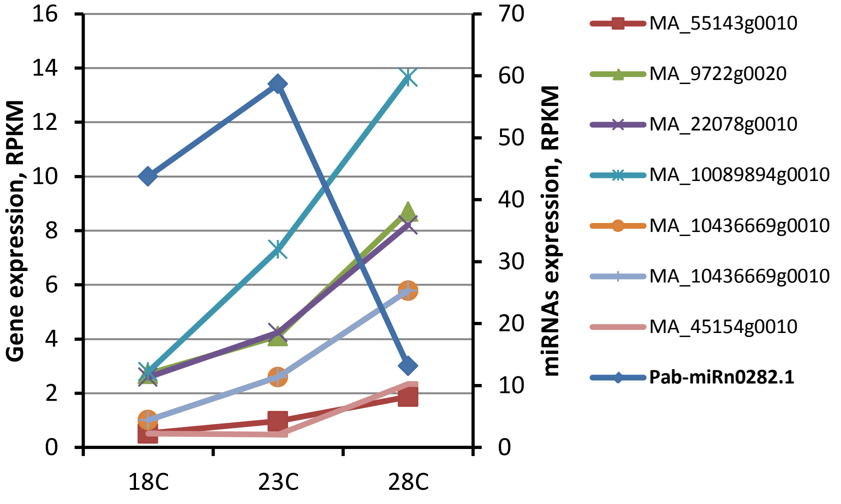	MA_55143g0010	PF00560 - Leucine Rich Repeat; PF00931 - NB-ARC domain; PF01637 - Archaeal ATPase; PF08937 - MTH538 TIR-like domain (DUF1863); PF12799 - Leucine Rich repeats (2 copies); PF12826 - Helix-hairpin-helix motif
		MA_9722g0020	PF00931 - NB-ARC domain; PF01582 - TIR domain; PF01637 - Archaeal ATPase; PF01926 - 50S ribosome-binding GTPase; PF03205 - Molybdopterin guanine dinucleotide synthesis protein B
		MA_22078g0010	PF00931 - NB-ARC domain; PF01582 - TIR domain; PF01637 - Archaeal ATPase; PF03205 - Molybdopterin guanine dinucleotide synthesis protein B; PF03308 - ArgK protein; PF05729 - NACHT domain
		MA_10089894g0010	PF00931 - NB-ARC domain; PF01582 - TIR domain; PF03205 - Molybdopterin guanine dinucleotide synthesis protein B; PF13191 - AAA ATPase domain; PF05729 - NACHT domain;
		MA_10436669g0010	PF01582 - TIR domain; PF02492 - CobW/HypB/UreG, nucleotide-binding domain; PF03205 - Molybdopterin guanine dinucleotide synthesis protein B; PF08937 - MTH538 TIR-like domain (DUF1863); PF13086 - AAA domain
		MA_45154g0010	PF01582 - TIR domain; PF13676 - TIR domain

20.	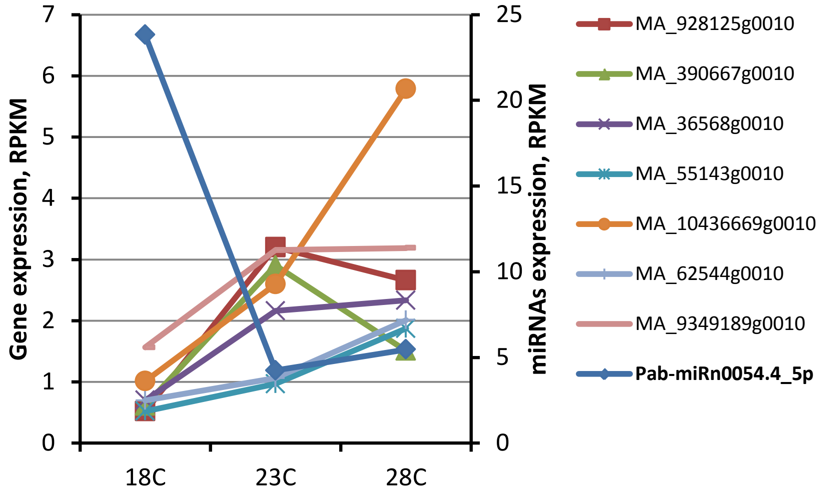	MA_928125g0010<br><br>MA_390667g0010<br><br>MA_36568g0010<br><br>MA_55143g0010<br><br>MA_10436669g0010<br><br>MA_62544g0010<br><br>MA_9349189g0010	PF00004 - ATPase family associated with various cellular activities (AAA); PF00931 - NB-ARC domain; PF01582 - TIR domain; PF01926 - 50S ribosome-binding GTPase; PF12799 - Leucine Rich repeats (2 copies); PF13676 - TIR domain<br>PF00931 - NB-ARC domain; PF01582 - TIR domain; PF03205 - Molybdopterin guanine dinucleotide synthesis protein B; PF05729 - NACHT domain; PF13191 - AAA ATPase domain; PF13676 - TIR domain<br>PF00560 - Leucine Rich Repeat; PF00931 - NB-ARC domain; PF01582 - TIR domain; PF03205 - Molybdopterin guanine dinucleotide synthesis protein PF13855 - Leucine rich repeat<br>PF00560 - Leucine Rich Repeat; PF00931 - NB-ARC domain; PF01582 - TIR domain; PF12826 - Helix-hairpin-helix motif; PF13306 - Leucine rich repeats (6 copies)<br>PF01582 - TIR domain; PF02492 - CobW/HypB/UreG, nucleotide-binding domain; PF03205 - Molybdopterin guanine dinucleotide synthesis protein B; PF13191 - AAA ATPase domain; PF13676 - TIR domain<br>PF00560 - Leucine Rich Repeat; PF00931 - NB-ARC domain; PF01582 - TIR domain ; PF13191 - AAA ATPase domain; PF13401 - AAA domain; PF13504 - Leucine rich repeat<br>PF00931 - NB-ARC domain; PF01582 - TIR domain; PF01926 - 50S ribosome-binding GTPase; PF13191 - AAA ATPase domain; PF13676 - TIR domain
