# Supplementary material for: In Silico Analysis of Small RNAs Suggest Roles for Novel and Conserved miRNAs in the Formation of Epigenetic Memory in Somatic Embryos of Norway Spruce
Source: Front Physiol. 2017 Sep 8;8:674. doi: 10.3389/fphys.2017.00674 (PMC5596105; doi:10.3389/fphys.2017.00674)
Supplement: Supplementary file 13 [file Image4.pdf]

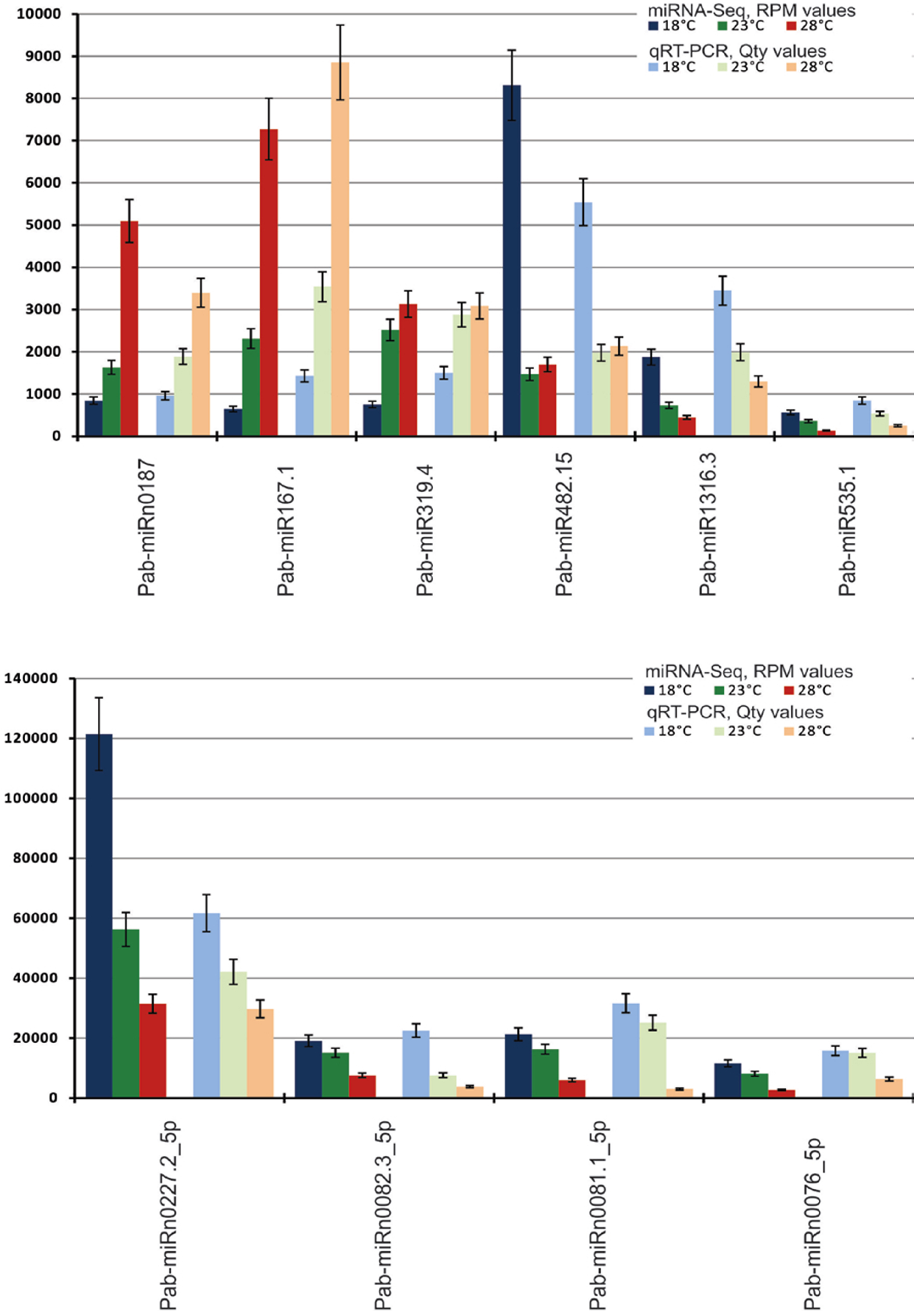

Suppl. figure S4. qRT-PCR validation for 10 differential expressed miRNAs, identified by miRNA-Seq in Norway spruce embryos developed under different epitype-inducing temperatures (18, 23 and 28°C). All data were averaged for 3 developmental stages, considered as biological replicates
